# A global network of biomedical relationships derived from text

**DOI:** 10.1093/bioinformatics/bty114

**Published:** 2018-02-27

**Authors:** Bethany Percha, Russ B Altman

**Affiliations:** 1 Biomedical Informatics Training Program, Stanford University, Stanford, CA, USA; 2 Department of Genetics and Genomic Sciences, Icahn School of Medicine at Mount Sinai, New York City, NY, USA; 3 Department of Bioengineering, Stanford University, Stanford, CA, USA; 4 Department of Genetics, Stanford University, Stanford, CA, USA; 5 Department of Medicine, Stanford University, Stanford, CA, USA

## Abstract

**Motivation:**

The biomedical community’s collective understanding of how chemicals, genes and phenotypes interact is distributed across the text of over 24 million research articles. These interactions offer insights into the mechanisms behind higher order biochemical phenomena, such as drug-drug interactions and variations in drug response across individuals. To assist their curation at scale, we must understand what relationship types are possible and map unstructured natural language descriptions onto these structured classes. We used NCBI’s PubTator annotations to identify instances of chemical, gene and disease names in Medline abstracts and applied the Stanford dependency parser to find connecting dependency paths between pairs of entities in single sentences. We combined a published ensemble biclustering algorithm (EBC) with hierarchical clustering to group the dependency paths into semantically-related categories, which we annotated with labels, or ‘themes’ (‘inhibition’ and ‘activation’, for example). We evaluated our theme assignments against six human-curated databases: DrugBank, Reactome, SIDER, the Therapeutic Target Database, OMIM and PharmGKB.

**Results:**

Clustering revealed 10 broad themes for chemical-gene relationships, 7 for chemical-disease, 10 for gene-disease and 9 for gene–gene. In most cases, enriched themes corresponded directly to known database relationships. Our final dataset, represented as a network, contained 37 491 thematically-labeled chemical-gene edges, 2 021 192 chemical-disease edges, 136 206 gene-disease edges and 41 418 gene–gene edges, each representing a single-sentence description of an interaction from somewhere in the literature.

**Availability and implementation:**

The complete network is available on Zenodo (https://zenodo.org/record/1035500). We have also provided the full set of dependency paths connecting biomedical entities in Medline abstracts, with associated sentences, for future use by the biomedical research community.

**Supplementary information:**

Supplementary data are available at *Bioinformatics* online.

## 1 Introduction

The network of interactions among biomedical entities—chemicals, genes and phenotypes—has long been of interest to biomedical researchers. Over the years, scientific curators have painstakingly excavated these relationships from the unstructured text of research articles, translating natural language descriptions into structured, machine-computable data. Manual curation provides researchers and clinicians with cross-sectional, domain-specific views of the literature and is responsible for such valuable resources as PharmGKB (Whirl-Carrillo, 2012), OMIM (Hamosh, 2005) and DrugBank (Wishart, 2006). Structured relationships offer insights into the mechanisms behind important higher-order relationships, such as drug-drug interactions (Percha and Altman, 2013; Swanson, 1986b). Combining them with an appropriate inferential model can also generate predictions of entirely new relationships, an approach called ‘literature-based discovery’ (Cohen and Hersh, 2005; Simpson and Demner-Fushman, 2012; Swanson, 1986a; Zweigenbaum *et al.*, 2007). As the literature grows, however, manual curation becomes increasingly time-consuming and expensive. It also limits the nature of questions we can ask of the literature to those for which substantial personnel and funding are available.

Curation maps diverse natural language descriptions onto a set of discrete, structured categories. Recent work in natural language processing has led to a class of methods, falling under the umbrella term ‘distributional semantics’, that perform a similar task using large corpora in place of human experts: these methods assess the semantic similarity of various terms by examining how they are used in context (Mikolov, 2013a; Turney and Pantel, 2010). Clustering words and phrases based on distributional similarity groups terms with similar meaning (Baker and McCallum, 1998; Cohen and Widdows, 2009), a feature that parallels the basic semantic mapping curators perform in their minds. Although most distributional semantics algorithms focus on words and phrases, they have also been applied to relationships (Section 2.1).

In earlier work (Percha and Altman, 2015), we showed that a distributional semantics algorithm called EBC could be combined with hierarchical clustering to derive clusters of drug-gene pairs that were related in similar ways. In this paper, we apply the same algorithm to cluster textual *descriptions* into classes, grouping descriptions of chemical-gene, gene–gene, gene-phenotype and chemical-phenotype relationships into ‘themes’. We then map thousands of natural language descriptions to one or more of these themes, including a quantitative score that represents the strength of the mapping. The result is a labeled, weighted network of biomedical relationships for all Medline abstracts. We compare our themes to known relationships from several biomedical databases, and provide the full network on Zenodo (https://zenodo.org/record/1035500).

## 2 Background

### 2.1 Biomedical relation extraction and curation

Biomedical relation extraction has a long history (Buyko, 2012; Chang and Altman, 2004; Coulet, 2010; Giuliano, 2006; Liu, 2016; Segura-Bedmar, 2011; Singhal, 2016), and many authors have suggested that the automated extraction of structured relationships from the literature can expedite database curation (Alex, 2008; Yeh 2003). However, relation extraction typically begins with a predefined schema: a set of one or more relationship classes onto which natural language descriptions are mapped. Sometimes the focus is simply to learn *whether* a particular sentence describes a relationship or not (Mallory, 2015). Our approach uses distributional semantics both to learn a schema and to map diverse surface forms onto relational classes. It draws from ideas in biomedical ontology learning (Liu, 2011) and entailment recognition as well as relation extraction.

### 2.2 Distributional semantics for relation extraction

While word models have dominated the distributional semantics literature (Deerwester, 1990; Mikolov, 2013a), distributional approaches have also been used to build representations of longer stretches of text such as phrases (Cho, 2014; Mikolov, 2013b; Passos, 2014), sentences (Kim, 2014), and documents (Le and Mikolov, 2014). Importantly, they have also been used to model relationships *between* pairs of entities, a type of similarity (‘relational similarity’) distinct from properties of the entities themselves (Levy, 2015; Turney and Pantel, 2010).

Several papers in the distributional semantics literature have examined relational similarity outside the biomedical domain (Dagan, 2013; Lin and Pantel, 2001; Riedel, 2013; Turney, 2005). Models that assess relational similarity typically operate on a matrix where the rows are pairs of entities (e.g. drug-gene pairs, chemical-gene pairs) and the columns are patterns that connect the entity pairs in the text (Section 2.4). Different methods may focus on the rows, columns, or both. Some cluster patterns in the text to discover groups of entity pairs that are related in similar ways (Hasegawa, 2004; Rosenfeld and Feldman, 2007; Shinyama and Sekine, 2006; Zhang, 2005), while others use the entity pairs to group the patterns (Lin and Pantel, 2001). Some methods, like EBC (Section 2.3), group both patterns and entity pairs at once (Bollegala, 2010; Kok and Domingos, 2008; Riedel, 2013; Yao *et al.*, 2011).

### 2.3 Ensemble Biclustering for Classification

Ensemble Biclustering for Classification (EBC) is a published distributional semantics method based on ensemble biclustering (Dhillon *et al.*, 2003; Percha and Altman, 2015) that has been shown to identify biomedical entity pairs expressing a certain type of relationship based on very few examples. It can be applied in an unsupervised fashion to generate a semantic ‘distance’ for the relationships between any two pairs of entities, similar to the cosine distance between word vectors in (Mikolov, 2013a). Combining EBC with hierarchical clustering produces a dendrogram of entity pairs that, at least in the case of drug-gene pairs, separates into recognizable groups (proteins that metabolize drugs, drugs that inhibit proteins, etc.; Percha and Altman, 2015). Because EBC is symmetric with respect to entity pairs and patterns, it can also be applied to cluster the patterns themselves, an approach we follow in this paper.

### 2.4 Dependency paths as patterns

**Fig. 1.**
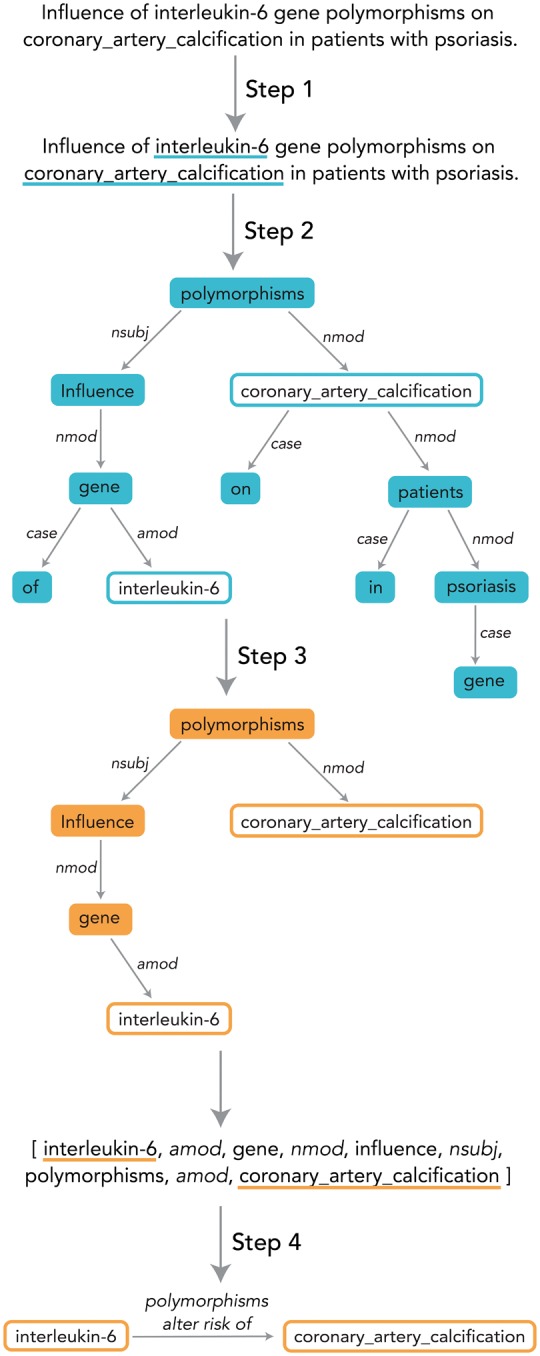
Process of converting a sentence to a structured relationship. Step 1: Named entity recognition. Step 2: Dependency parsing to produce dependency graph. Step 3: Dependency path extraction from dependency graph. Step 4: Mapping of dependency path to relationship data structure, which consists of the two entities, a direction and a structured ‘theme’ that reflects the nature of the relationship between the two entities. The methods in this paper focus on Step 4

Applying EBC, or indeed any distributional semantics algorithm, to assess relational similarity requires us to define what constitutes a ‘pattern’ that connects pairs of entities in the text. For our purposes, a pattern is a structure called a *dependency path*. Dependency paths are produced automatically using the Stanford dependency parser (De Marneffe and Manning, 2008a). The input to the parser is a raw Medline sentence, and the output is a dependency graph. A dependency graph (Fig. 1) is one way to represent the grammatical architecture of a sentence; the nodes are words, and the edges are grammatical dependencies (grammatical relationships between pairs of words, described in detail in De Marneffe and Manning, 2008b).

A dependency path is a path through a dependency graph that connects two entities. Focusing on the dependency path helps prune out irrelevant terms and phrases and focus the algorithm’s attention on the part of the sentence directly relevant to the relationship between the two entities. It is possible for a single sentence to generate multiple dependency paths if more than two entity names are present in the sentence.

## 3 Materials and methods

### 3.1 Named entity recognition using PubTator

The NCBI project PubTator (Wei, 2013) provides high-quality named entity annotations of (1) drugs and other chemicals, (2) genes and proteins and (3) diseases, side effects and other phenotypes for all of Medline. Following PubTator’s convention, we use the categories ‘chemical’, ‘gene’ and ‘disease’ to refer to groups 1, 2 and 3, respectively. PubTator annotations for a single abstract consist of the full text of the abstract, its title and a series of annotated concepts for which it provides: the location and string in the raw text that matched the concept, its entity type (chemical, gene, disease, etc.) and its closest database identifier. There are approximately 16.5 million Medline abstracts annotated by PubTator as of this writing. Annotations are updated monthly. Our version of the PubTator annotations was downloaded on April 30, 2016.

### 3.2 Extraction of dependency paths

We used the PubTator annotations to concatenate phrases corresponding to annotated biomedical entities; for example, the phrase ‘cytochrome p450 3A4’, if identified as an entity by PubTator, was changed to ‘cytochrome_p450_3A4’ (using the underscore). We then divided the annotated and concatenated abstracts into sentences and parsed each sentence using the Stanford Dependency Parser (De Marneffe and Manning, 2008a) to produce a dependency graph. From there, we found the dependency paths connecting (a) chemicals to genes, (b) chemicals to diseases, (c) genes to diseases, (d) genes to genes, using the method in Figure 1.

The extraction of the gene–gene paths introduced an additional layer of complexity, since there is no natural way to orient the paths. We therefore extracted two paths for a dependency graph connecting two genes G1 and G2: the path from G1 to G2, and the path from G2 to G1.

As in (Percha and Altman, 2015), we eliminated paths containing dependencies of type *conj* (two elements connected by a coordinating conjunction; De Marneffe and Manning, 2008b), because these were usually errors arising from how the dependency parser represents lists.

### 3.3 Creating the data matrices

We selected the most frequent ∼700 dependency paths connecting (a) chemicals to genes, (b) chemicals to diseases, (c) genes to diseases, (d) genes to genes and sampled 2000 entity pairs from the total set connected by one or more of those paths. We reasoned that we needed enough different dependency paths to capture the diversity of potential themes, but not so many that the resultant dendrograms would be too large for manual review.

We arranged the data in matrices in which the rows were entity pairs and the columns were dependency paths. There was a ‘1’ in matrix cell *ij* if dependency path *j* connected entity pair *i* somewhere in Medline, and a ‘0’ if not. Descriptions of the four matrices can be found in Table 1.

**Table 1.** Descriptions of data matrices for all four interaction types

Type	Dependency paths	Minimum path occur.	Nonzero elements	Row clusters (*K*)	Column clusters (*L*)
Chemical-gene	697	5	6276	100	100
Chemical-disease	636	5	6022	150	170
Gene-disease	739	12	6450	190	150
Gene–gene	693	100	7903	90	70

*Note*: Each contained 2000 entity pairs.

### 3.4 Applying EBC and hierarchical clustering

We found optimal row and column cluster numbers (*K* and *L* in Table 1) for EBC using the heuristic described in (Percha and Altman, 2015). Using the optimal *K* and *L*, we applied EBC (Section 2.3) to the four matrices, performing the biclustering 2000 times and recording the number of times each column (dependenc path) clustered with every other column. We followed the technique described in (Percha and Altman, 2015) to convert this array of coclustering frequencies into a correlation matrix and applied hierarchical clustering with minimax linkage (Bien and Tibshirani, 2011) to produce dendrograms. The major difference between our approach here and that in (Percha and Altman, 2015) is that here the dendrogram leaves are dependency paths, and in that paper they were drug-gene pairs. We produced one dendrogram for each of four relationship types: chemical-gene, chemical-disease, gene-disease and gene–gene.

### 3.5 Cluster theme labeling

We cut the four dendrograms at a level that produced 30 clusters. Any clusters of 10 or fewer dependency paths were not examined further, and upon visual inspection of the dendrograms, very large clusters with obvious internal structure were cut further down to produce smaller subclusters. For each cluster, a set of 10 dependency paths was selected at random and a human annotator examined the paths and several associated example sentences from the literature to assign a label. Nearby clusters sometimes shared similar themes, so we simplified the clusters into thematically-related groups and assigned each theme a symbol. Supplementary Material contains the complete set of intermediate labels and sample dependency paths for each cluster.

### 3.6 Assigning remaining paths to themes

The themes derived from the dendrograms are based on only the most frequent ∼700 dependency paths (Section 3.4). We call these the *flagship paths* for each theme. However, there are vastly more dependency paths than this in the full dataset. To assign the remaining paths to themes, we counted the number of times each path co-occurred with the flagship paths for each theme. A co-occurrence is a situation where both the unassigned path and a flagship path connect the same entity pair. We calculated co-occurrence frequencies for the flagship paths as well as the non-flagship paths. We refer to the number of co-occurrences of each path with flagship paths for a particular theme as that path’s *support* for that theme.

### 3.7 Evaluating against database relations

We used known relationships from six human-curated databases to evaluate the validity of our themes. The evaluation databases included DrugBank (Wishart, 2006), PharmGKB (Whirl-Carrillo, 2012), the Therapeutic Target Database (TTD; Zhu, 2011), SIDER (Kuhn, 2015), OMIM (Hamosh, 2005) and Reactome (Croft, 2010). In all six cases, interactions were converted from database identifiers to strings using whatever synonym mapping files were available from each database, if any. Strings were lowercased and multi-word terms were concatenated using the underscore, and these lists were then filtered against our dataset of co-occurring entities from PubTator.

Table 2 contains information about the databases we used and the number and type of relationships pulled from each. In the case of SIDER, we used UMLS to find the set of all strings corresponding to each drug concept identifier but did not use synonyms for disease names beyond what was reported in SIDER itself. We also restricted drug side effects to those with an occurrence frequency >30% in SIDER. For Reactome, we queried the UniProt API to convert its native UniProt protein identifiers to strings. We used the PharmGKB relationships file to find gene–gene pathway interactions and also considered gene-disease interactions (‘association’ in Table 2) despite the fact that PharmGKB gene-disease associations can include both the obvious causal mutations as well as situations in which a polymorphism in a gene impacts response to a drug used to treat a disease, thus leading to a gene-disease association. DrugBank gene–gene associations were those drug-target associations for which the ‘chemical’ was actually a protein.

**Table 2.** Databases and associated relationship types used for evaluation

Type	Database	Relation type	Count
Chemical-gene	DrugBank	Drug-target	619
	TTD	Metabolism	286
		Transport	143
		Inhibition	195
		Agonism	40
		Antagonism	43
Chemical-disease	SIDER	Side effect	521
	TTD	Indication	1611
		Indication	1234
		Biomarkers	52
Gene-disease	PharmGKB	Association	375
	TTD	Disease target	688
	OMIM	Causal mutation	918
Gene–gene	PharmGKB	Pathway	147
	Reactome	Protein complex	216
	DrugBank	Protein-target	38

*Note*: The relationship counts are the numbers of entity pairs (represented as strings, not database identifiers) found in the database that also occurred in our dataset.

**Fig. 2.**
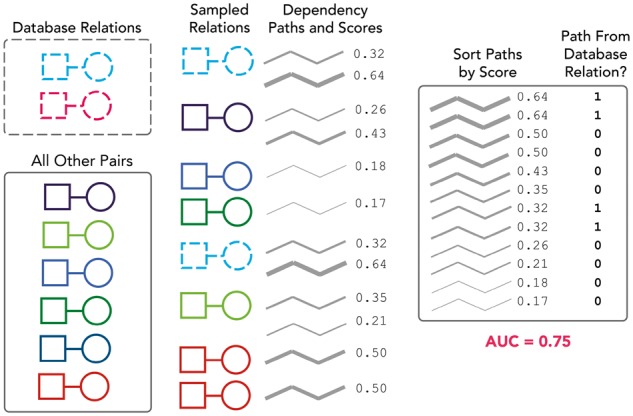
Evaluation against known database relations. In this example, the squares represent diseases, the circles represent genes and we are evaluating one particular gene-disease theme. The database contains two relations (gene-disease pairs) that also appear in our dataset (i.e. co-occurred in a sentence at least once, connected by a dependency path to which theme supports could be assigned). There are also six other gene-disease pairs in our dataset that are not found in the database; these serve as our negative ‘background’. We create 100 bootstrap samples by sampling with replacement from both the database and background sets (only a single sample is shown here). We rank all dependency paths that connect our sampled entity pairs based on their supports for the theme. Note that the scores here are fractions and not the raw supports because we normalize the supports across all themes (by dividing by the total support across all themes) so as not to disadvantage less common dependency paths. We then calculate an AUC for the ranking against labels representing whether the entity pair connected by the path was a known database relation (1) or not (0). We repeat this process across all 100 samples and calculate a mean and standard deviation for the AUC

Figure 2 shows a summary of our evaluation process. We sought to evaluate our theme assignments, but themes are assigned to *dependency paths*, whereas the database relations are *entity pairs* (a single entity pair can have multiple dependency paths corresponding to multiple sentences). We therefore evaluated the degree to which ranking the dependency paths by their supports for a particular theme would cause the paths connecting known database pairs to filter to the top (see Fig. 2 caption). Our evaluation metric for each theme was the AUC of the ranked lists of dependency paths, averaged across 100 bootstrap replicates.

We considered a positive outcome to be a situation in which (a) one or a few themes produced significantly higher AUCs than others, and (b) the themes producing the highest AUCs corresponded to the type of relationship(s) contained in the database. Negative outcomes included situations in which no theme produced a better AUC than any other, as well as situations where a non-corresponding theme ranked the database relationships more highly (e.g. ranking chemical-disease dependency paths by their supports for the ‘side effect’ theme would mysteriously cause drug-indication pairs to filter to the top).

## 4 Results

### 4.1 Four dendrograms

The dendrograms for all four relationship types are shown in Figure 3a (chemical-gene), Figure 3b (chemical-disease), Figure 3c (gene-disease) and Figure 3d (gene–gene), along with sample dependency paths from a few of the major clusters. Full descriptions of all of the clusters with descriptions and dependency paths can be found in the Supplementary Material.

**Fig. 3.**
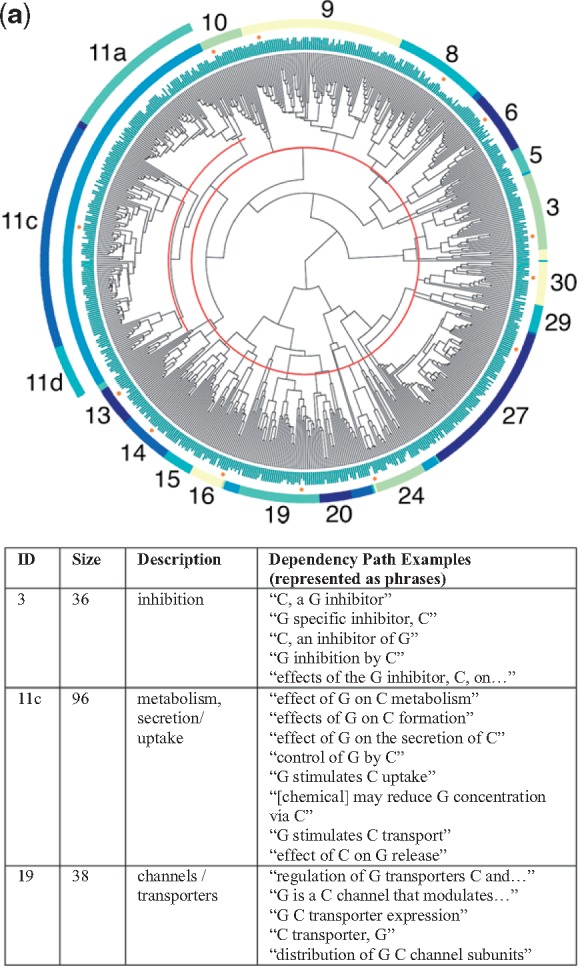
(**a**) Chemical-gene dendrogram. Each leaf node represents one dependency path. In the example patterns above, C represents the chemical and G the gene/protein

**Fig. 3.**
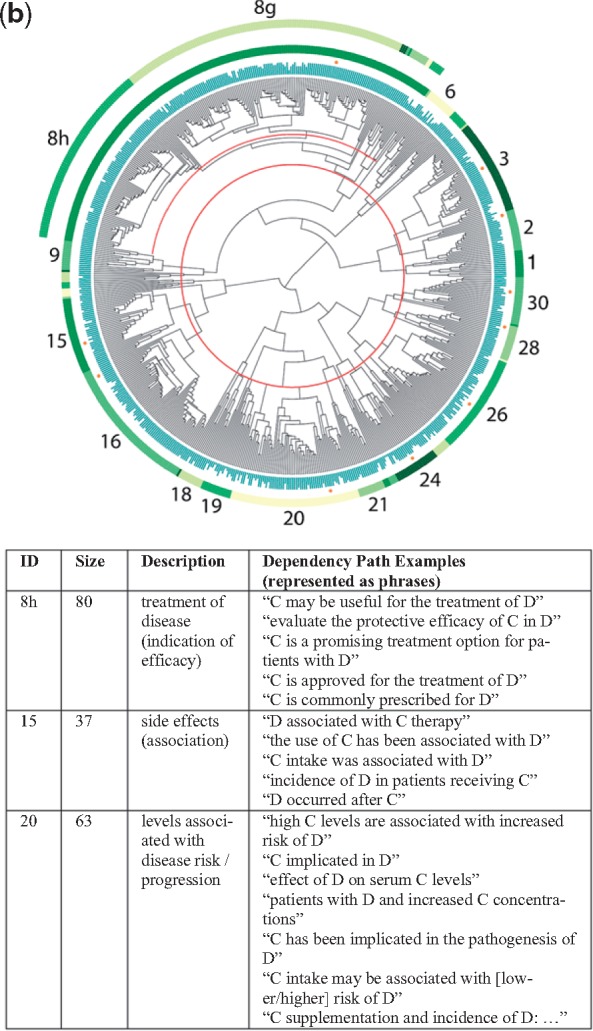
(**b**) Chemical-disease dendrogram. Each leaf node represents one dependency path. In the example patterns above, C represents the chemical and D the disease/phenotype

**Fig. 3.**
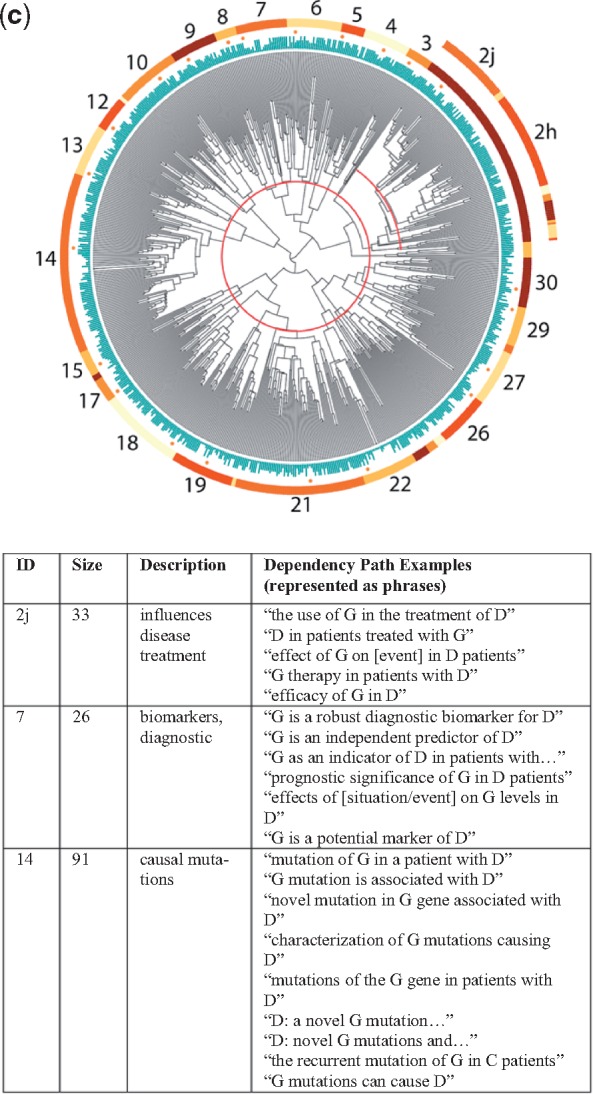
(**c**) Gene-disease dendrogram. Each leaf node represents one dependency path. In the example patterns above, G represents the gene/protein and D the disease/phenotype

**Fig. 3.**
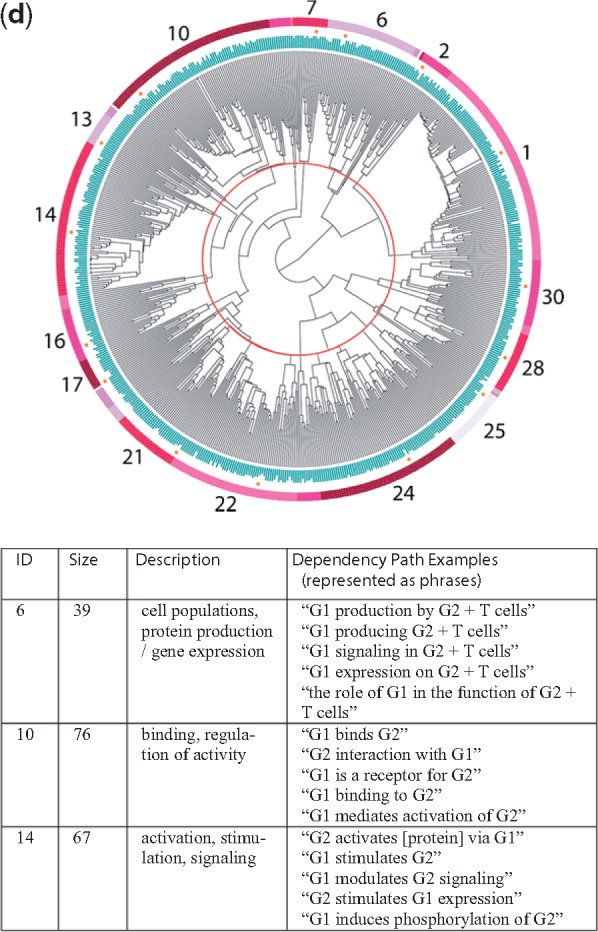
(**d**) Gene–gene dendrogram. Each leaf node represents one dependency path. In the example patterns above, G1 represents the first gene/protein and G2 the second gene/protein

### 4.2 Simplified relationship themes

Table 3 contains the complete list of themes for each of the four relationship types. Two of the groups in the chemical-gene dendrogram contained relationships where we perceived the directionality to be important for future applications: activation (agonism versus antagonism; cluster 6) and changes in expression (up, down or neutral; clusters 8–10). The clusters were small enough that we decided to label the positive and negative directional dependency paths manually to ensure perfect separation; this is what the ‘+’ and ‘–’ signs refer to in Table 3.

**Table 3.** Simplified relationship themes derived from the clusters shown in Figure 3a–d

Type	Symbol	Theme	Relevant figure	Supporting cluster(s)
Chemical-gene	A+	Agonism, activation	3a	6+
	A−	Antagonism, blocking		6–
	**B**	Binding, ligand (esp. receptors)		14–16
	**E+**	Increases expression/production		8+, 9+
	E−	Decreases expression/production		8–, 9–, 10
	**E**	Affects expression/production (neutral)		8, 9, 11a
	N	Inhibits		3
Gene-chemical	O	Transport, channels	3a	19, 21
	K	Metabolism, pharmacokinetics		11c
	Z	Enzyme activity		20
Chemical-disease	T	Treatment/therapy (incl. investigatory)	3b	8g, 8h, 9
	C	Inhibits cell growth (esp. cancers)		2, 3
	Sa	Side effect/adverse event		6, 15, 16
	Pr	Prevents, suppresses		1, 9, 21, 24, 28
	Pa	Alleviates, reduces		26, 30
	**J**	Role in pathogenesis		20
Disease-chemical	Mp	Biomarkers (progression)	3b	18, 19
gene-disease	U	Causal mutations	3c	14
	Ud	Mutations affect disease course		13
	D	Drug targets		10, 12
	**J**	Role in pathogenesis		2h, 4, 6, 8, 9
	Te	Possible therapeutic effect		2j, 3
	Y	Polymorphisms alter risk		22, 26, 27
	G	Promotes progression		29
Disease-gene	Md	Biomarkers (diagnostic)	3c	5, 7
	X	Overexpression in disease		15, 17, 30
	L	Improper regulation linked to disease		18, 19, 21
Gene–gene	**B**	Binding, ligand (esp. receptors)	3d	10
	W	Enhances response		13
	V+	Activates, stimulates		14, 16
	**E+**	Increases expression/production		21, 22
	**E**	Affects expression/production (neutral)		7, 17
	I	Signaling pathway		24
	H	Same protein or complex		25
	Rg	Regulation		28, 30
	Q	Production by cell population		1, 2, 6

*Note*: A symbol is bolded if it refers to a theme that appears in multiple dendrograms. Complete descriptions of the individual clusters can be found in the Discussion. Examples of dependency paths from each cluster are in the Supplementary Material for this article.

As expected, nearby clusters sometimes reflected similar themes. Occasionally, clusters that were not close together in the dendrograms also shared similar themes. This most often occurred when the same relationship type was described in slightly different ways within distinct groups of entity pairs. For example, clusters 6, 15 and 16 in Figure 3b all referred to descriptions of side effects or adverse events related to the administration of a chemical, yet cluster 6 was also closely related to clusters 8 and 9, which described investigations of experimental agents.

### 4.3 Evaluation of themes using known database relations

Figure 4 shows the results of our evaluation against the human-curated database relations described in Table 2. We consider a theme enriched if the mean AUC for that theme was more than one standard deviation above 0.5 across 100 bootstrap replicates.

**Fig. 4.**
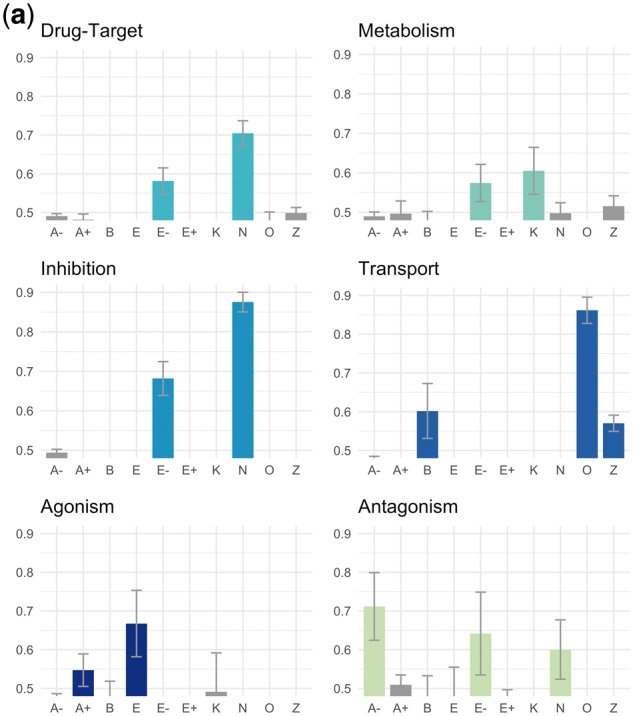
(**a**) Chemical-gene theme evaluations. This caption refers to (a)–(d). In all cases, the *y*-axis refers to AUC for ranking dependency paths connecting known database relations against others using scores based on their supports for a given theme (Fig. 2). Descriptions of the theme symbols are in Table 3. Error bars are one standard deviation of AUC across 100 bootstrap replicates. A bar is colored if the mean AUC is >1 SD above 0.5. Some themes led to AUCs <0.5 (i.e. database relations were depleted for these themes instead of enriched) and were cut off because the *y*-axis starts at 0.5

**Fig. 4.**
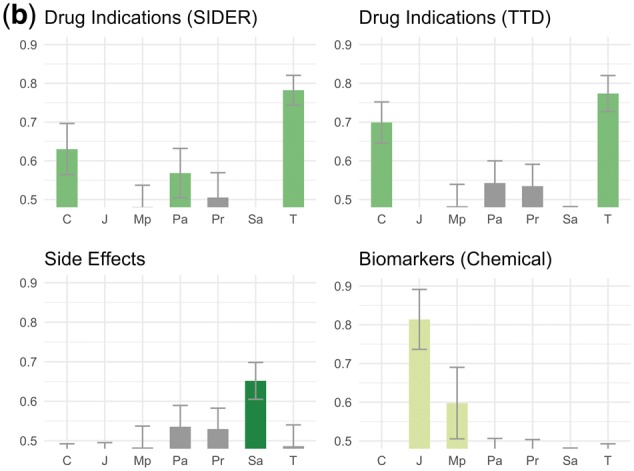
(**b**) Chemical-disease theme evaluations. See caption (a)

**Fig. 4.**
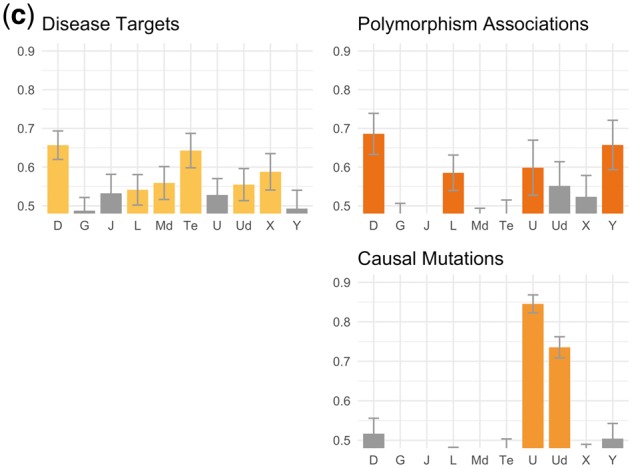
(**c**) Gene-disease theme evaluations. See caption in (a)

**Fig. 4.**
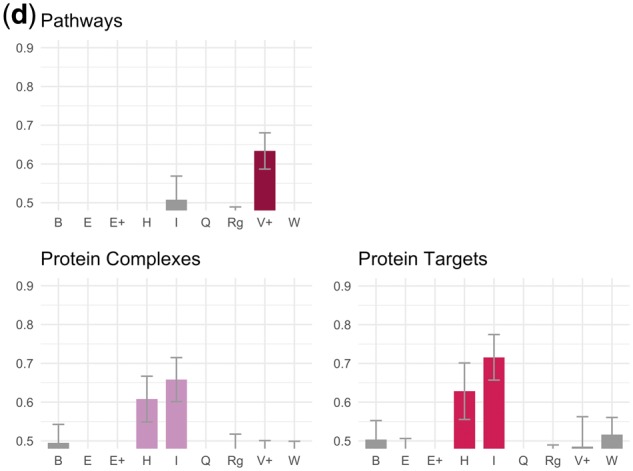
(**d**) Gene–gene theme evaluations. See caption in (a)

In Figure 4a (chemical-gene), both drug-target and inhibition database relations were enriched for the *N* (inhibition) and *E-* (decreased expression) themes (Table 3) and no others. Since drugs administered to target a particular protein are often inhibitors of that protein, this consistency makes sense (note: only 71 drug-gene pairs overlapped between the DrugBank ‘drug-target’ and TTD ‘inhibitor’ datasets, so this was not just due to overlapping entity pairs in the two datasets). Known transport relations from DrugBank were enriched for themes *O* (transport), *B* (binding) and *Z* (enzyme activity) with the strongest enrichment for theme *O*. Agonism versus antagonism relations from the same database (TTD) displayed no overlapping enriched themes. Agonism relations were enriched for theme *A+ *(agonism) and *E* (affect on expression), whereas antagonism relations were enriched for theme *A-* (antagonism), *E-* (decreased expression) and *N* (inhibition). Metabolism relations, which we obtained from the DrugBank ‘enzymes’ field, were enriched for the *K* (metabolism/pharmacokinetics) theme, as well as the *E-* theme.

In Figure 4b (chemical-disease), drug indications and side effects from the same database (SIDER) showed opposing patterns of enrichment. Indication relations were enriched for the *T* (treatment), *C* (cancer treatment) and *Pa* (prevention and alleviation of symptoms) themes, while side effect relations were only enriched for the *Sa* (side effect/adverse event) theme. The pattern of enrichment for drug indication relations was repeated when we used the TTD database (178 overlapping relations with SIDER, representing 14% of the smaller dataset), with the exception of the *Pa* theme, which was not enriched. Biomarkers were enriched for the *J* (role in pathogenesis) and *Mp* (biomarkers) themes and none of the side effect or treatment themes.

In Figure 4c (gene-disease), the known causal/pathogenic mutation relationships extracted from OMIM were strongly enriched for the *U* (causal mutation) and *Ud* (mutation affecting disease prognosis) themes and no others. The disease target relationships from TTD and the gene-disease associations from PharmGKB were each enriched for several themes. Disease targets from TTD, which describe situations where a protein is being targeted for treatment of a disease, were enriched for themes *D* (drug target), *Te* (possible therapeutic effect by targeting protein), *L* (improper regulation linked to disease), *Md* (diagnostic biomarkers), *X* (overexpression in disease) and *Ud* (mutation affecting disease prognosis). While the PharmGKB relations shared enrichment for themes *D* and *L*, they had no enrichment for the other TTD themes but were enriched for themes *U* (causal mutations) and *Y* (polymorphisms altering risk).

In Figure 4d (gene–gene), database relations involving protein complexes (Reactome) and proteins targeting other proteins (DrugBank) were both enriched for the same two themes: *H* (protein complexes) and *I* (signaling pathways). The relations from PharmGKB, which describe situations where two proteins are part of a pharmacokinetic or pharmacodynamics pathway for a drug, were enriched for theme *V+ *(activation/stimulation) and no other themes.

### 4.4 Description of the final network

Our final dataset is a network with labeled, weighted edges. Because a single dependency path can support multiple themes, the network is a multigraph.

The full network is available in two parts on Zenodo (https://zenodo.org/record/1035500).

Part I connects dependency paths to themes. Each record contains a dependency path followed by the supports for each theme, and indicators for whether or not the path is part of the flagship path set for each theme.

Part II connects sentences to dependency paths. It consists of sentences and associated metadata, entity pairs found in the sentences and dependency paths connecting those entity pairs. Each record contains all of the information shown in Table 4. Part II contains 4 451 661 records, of which 92 465 (2.1%) represent chemical-gene dependency paths, 3 875 209 (87.1%) are chemical-disease paths, 338 306 (7.6%) are gene-disease paths and 145 681 (3.3%) are gene–gene paths. We have arranged the paths in alphabetical order of the entity pairs, so that different sentences referring to the same two entities appear next to each other in the file.

**Table 4.** Information in a single record from Part II of the final network dataset

Example	Description
15161679	PubMed ID
0	Sentence number (0 = title)
zosuquidar_trihydrochloride	First entity name, formatted
54, 81	First entity name, location
P-glycoprotein	Second entity name, formatted
28, 42	Second entity name, location
zosuquidar trihydrochloride	First entity name, raw string
P-glycoprotein	Second entity name, raw string
MESH: C095179	First entity, database id(s)
5243	Second entity, database id(s)
Chemical	First entity, type
Gene	Second entity, type
trial|appos|START_ENTITY trial|nmod|inhibitor inhibitor|amod|END_ENTITY	Dependency path
A Phase I trial of a potent P-glycoprotein inhibitor, zosuquidar_trihydrochloride –LRB- LY335979 –RRB-, administered intravenously in combination with doxorubicin in patients with advanced malignancy.	Sentence, tokenized

*Note*: The database IDs are from PubTator and correspond to the entity-type-specific databases described in Wei (2013).

### 4.5 A note on coverage

We were able to assign theme supports to 37 491 chemical-gene dependency paths (13.6% of total), 2 021 192 chemical-disease dependency paths (33.3%), 136 206 gene-disease dependency paths (20.0%) and 41 418 gene–gene dependency paths (11.0%). The rest of the dependency paths never co-occurred with a single flagship path for any theme, so we could not calculate theme supports for them.

However, since some dependency paths occur more frequently than others, our coverage at the sentence level is somewhat higher. If we consider all extractable relationship triples somewhere in the literature (two entities connected by a dependency path in a single sentence), we are able to assign themes to 92 465 chemical-gene connections out of 556 487 (16.6%). For chemical-disease connections, of which there are 13 658 821 in Medline, we can assign themes to 3 875 209 (28.4%). For gene-disease connections, we can assign themes to 338 306 out of 1 071 043 (31.6%), and for gene–gene, we can assign themes to 145 681 out of 1 274 010 (11.4%).

## 5 Discussion

### 5.1 Summary of our approach and its advantages

We have constructed a labeled, weighted network of structured biomedical relationships for all Medline abstracts. Inputs to our method were (a) the complete set of named entity annotations from PubTator and (b) human annotation of the clusters produced by EBC. The discovery of clusters of dependency paths corresponding to different classes of biomedical relations was handled by EBC and based solely on patterns in the text. In our approach, a theme is not just an abstract concept like ‘inhibits’; it is a set of dependency paths that tend to connect similar entities. Some of our themes may have been obvious to a human schema creator, but others were less obvious, and EBC often distinguishes themes that a human may have lumped together (‘mutations affecting disease course’ versus ‘causal mutations’) and combines themes that a human may have separated (directionality of expression changes, for example). We believe making theme decisions based on clusters of textual patterns represents a principled approach to schema creation.

Mapping other dependency paths to the schema is as simple as looking for co-occurrence of new paths with the flagship paths for the different themes. A single dependency path can provide support for multiple themes, so the themes can be reconfigured and new themes can be introduced at any time, without altering the supports for existing themes. As Medline grows, new data can be labeled quickly and seamlessly simply by considering co-occurrence frequencies with the flagship paths for different themes, as described earlier. The most difficult and time-consuming part of the process is the manual labeling of the clusters, and this need only be performed once.

### 5.2 An illustrative example

Unfortunately, due to space limitations, we needed to place the detailed descriptions of most of the different clusters in Figure 3 and Table 3 in the Supplementary Material, along with the sample dependency paths used in the manual labeling process. However, we present one example here to illustrate the power of this approach.

Consider a situation where we believe a particular genetic mutation will decrease the body’s ability to metabolize a certain drug. We may want to search the literature for all of the side effects that have ever been observed for that drug, so we can monitor those symptoms in patients with the mutation. Unfortunately, the vast majority of sentences in which a phenotype occurs with the drug describe treatment relationships, where the drug is being used to *treat* or *prevent* a certain condition and does not *cause* it. Unless we have extensive knowledge of the drug and its indications, sifting through hundreds of sentences to identify side effect relationships can be time consuming. Restricting our search to dependency paths with high support for theme Sa (Table 2) can help us prune away irrelevant data.

In addition, very few sentences in the literature actually say ‘D is a side effect of drug C’. However, clusters 15 and 16 in Figure 3b contain multiple different dependency paths indicating that idea, corresponding to patterns like ‘the use of C has been associated with D’, ‘C intake was associated with D’, ‘administration of C resulted in D’ and ‘patient developed D after receiving C’ (see Fig. 3b caption). The grouping of these various patterns, which contain diverse word choice and phrasing, occurred automatically during the clustering process. All of the sentences containing one of these patterns, along with dozens more just like them, would receive a high score for theme Sa. From a practical standpoint, thematic labeling represents a filtering process on the text of the literature that can help target literature searches for a variety of research and clinical needs.

### 5.3 Notes on evaluation

We used known biomedical database relations to evaluate our themes because they constitute the largest and most objective sources of structured biomedical relationships of interest to the scientific community. We observed two promising findings: (1) our themes often corresponded directly to relationship types captured in databases, and (2) in nearly all cases, the correct themes were enriched among database entity pairs. For example, drug indication themes (*T, C, Pa*) were enriched among drug indication pairs from SIDER, whereas the *Sa*, or side effect, theme was enriched among drug-side effect pairs from the same database. For OMIM, which tracks the relationships between genetic variants and their resultant phenotypes, the only enriched themes were for mutations causing disease or affecting its course. This means that if we were to rank natural language descriptions of gene-phenotype relationships by their scores on the *U* (causal mutations) theme, we would be more likely to prioritize descriptions of relationships appropriate for OMIM than if we were to rank them by their scores on some other theme. If we want to build a database like OMIM from scratch, we should consider not only gene-phenotype entity pairs that frequently co-occur in sentences; we should focus our attention on sentences reflecting this particular theme.

We did not begin evaluating our themes against biomedical databases until long after the network had been created, so we were encouraged to see strong enrichment for the appropriate themes in many cases. Aside from OMIM and its relationship to the *U* (causal mutations) theme, the *O* (transport) theme is strongly enriched for chemical-protein transport relationships and the *E-* (decreased expression) and *N* (inhibition) themes are strong indicators of drug-target and protein-inhibitor relationships. Protein–protein binding is indicated by the *H* (protein complexes) and *I* (signaling) themes, while chemical biomarkers for diseases can be found by looking for enrichment of the *J* (role in pathogenesis) and *Mp* (biomarker) themes.

### 5.4 Study limitations

However, our approach is not without limitations. Its most significant downside is that it relies on the co-occurrence of different dependency paths to map rarer paths to themes. There are a large number of dependency paths that (a) never co-occur with another path, and (b) occur with only one entity pair. These orphan paths cannot be assigned to themes using the current method (Section 4.5). In the future, addition of a pre-processing step that simplifies and unifies diverse dependency paths, such as Biosimplify (Jonnalagadda and Gonzalez, 2010), might help rescue some of these orphan paths. In addition, a dependency path can only capture the relationship between two entities in a sentence, but many relationships involve more than two entities. Right now, we miss these more complex relations.

Another issue with using dependency paths as patterns is the potential for parser error. In (Percha and Altman, 2015) we identified several cases where the parser’s construction of the dependency graph led to dependency paths that bypassed words relevant to the meaning of the relation. Avoiding this problem will likely mean incorporating additional features in addition to the dependency path, such as other dependencies in the sentence.

An issue particular to gene–gene relationships, or any type of symmetric relationship, is that our method treats each direction separately. We were interested to see whether the dendrogram in Figure 3d would fragment into two halves, each containing relationships of a particular directionality, but this did not occur. Many of the gene–gene relationships in Table 2 are symmetric (binding, for example), but at this time we are unable to distinguish directionality in, for example, activation relationships. We will investigate this issue, and all dependency path issues, further as we develop the next version of our network.

We initially intended to include chemical-chemical and disease-disease relations in our network in addition to the other four types. However, we observed that the majority of single-sentence co-occurrences for these types did not represent true relationships, and instead consisted of chemical pairs present in lists, for example, in addition to errors where, for example, a protein was tagged as a chemical. We may solve this problem in future versions of the network by applying a system like DeepDive (Mallory, 2015) as a first step to weed out sentences that are unlikely to contain true relations.

Finally, the named entity recognition provided by PubTator, while state-of-the-art, is not perfect. While the multi-word entity recognition provided by PubTator is a huge improvement over the simple lexicon matching NER used in (Percha and Altman, 2015), we have also observed several situations where only parts of entity names are captured, or where entities are assigned to the wrong type (proteins labeled as chemicals, etc.). However, we expect that NCBI will continue to refine its NER algorithms in the coming years.

### 5.5 Applications of the network and future directions

Our hope is that efficient schema creation and relationship extraction from the literature will enable faster search and organization of scientific findings by curators and researchers across a variety of disciplines. We particularly hope that domain experts can use our network to quickly and easily build up literature-based knowledge bases for new domains and to identify the specific sentences in the literature where relationships are described. For this reason, we are releasing the raw dependency paths and tokenized sentences from PubTator along with our thematically-labeled edges.

Structured networks like ours also enable new research directions. Each edge in this network represents one discovery, made by some scientist in a particular time and place. By combining them in a unified, structured format, we can start to look for network motifs (Milo, 2002) representing mechanisms for drug-drug interactions, characteristic patterns of pharmacokinetic interactions in drug metabolism pathways and genetic and chemical patterns underlying complex phenotypes.

## Funding

RBA is supported by National Institutes of Health [LM05652, GM061374, GM102365] and a gift from Oracle. B.P. was supported by a Morgridge Family Stanford Interdisciplinary Graduate Fellowship.

*Conflict of Interest*: none declared.

## Supplementary Material

Supplementary Data
