# Supplementary material for: A global network of biomedical relationships derived from text
Source: Bioinformatics. 2018 Feb 27;34(15):2614–24. doi: 10.1093/bioinformatics/bty114 (PMC6061699; doi:10.1093/bioinformatics/bty114)
Supplement: Supplementary Data [file bty114_bioinf-v3-supplementa.docx]

**Supplement A: Descriptions of the Four Dendrograms, with Examples**

*A.1 Chemical-gene relationships*

Here we describe the major clusters in Figure 2 of the main paper (reproduced below). We use "C" to represent a chemical, and "G" to represent a gene.

The first major cluster, cluster 3, refers to inhibition (the chemical, C, is an inhibitor of the protein, G). This is reported mostly in a static context in patterns such as “C, a G inhibitor” and “G inhibition by C”. The mechanism behind the inhibition is usually unclear from these descriptions. Is C inhibiting the activity of the protein G or the expression of G’s mRNA? It’s difficult to tell.

Clusters 5 and 6 specifically describe effects on protein activity with 6, the larger cluster, referring mainly to situations where C is an agonist or antagonist of G. Antagonists are often referred to as “blockers” or “inhibitors”, while agonists are referred to as “activators” or “ligands”.

Clusters 8, 9, 10 and 11a all describe effects on mRNA and protein levels, rather than protein activity. Cluster 10 specifically refers to inhibition, while the effects in clusters 8 and 9 are mixed: some positive, some negative, some neutral. Cluster 11a sometimes refers to a treatment response, as though C is administered as a therapy or the paper is investigating G’s response to C.


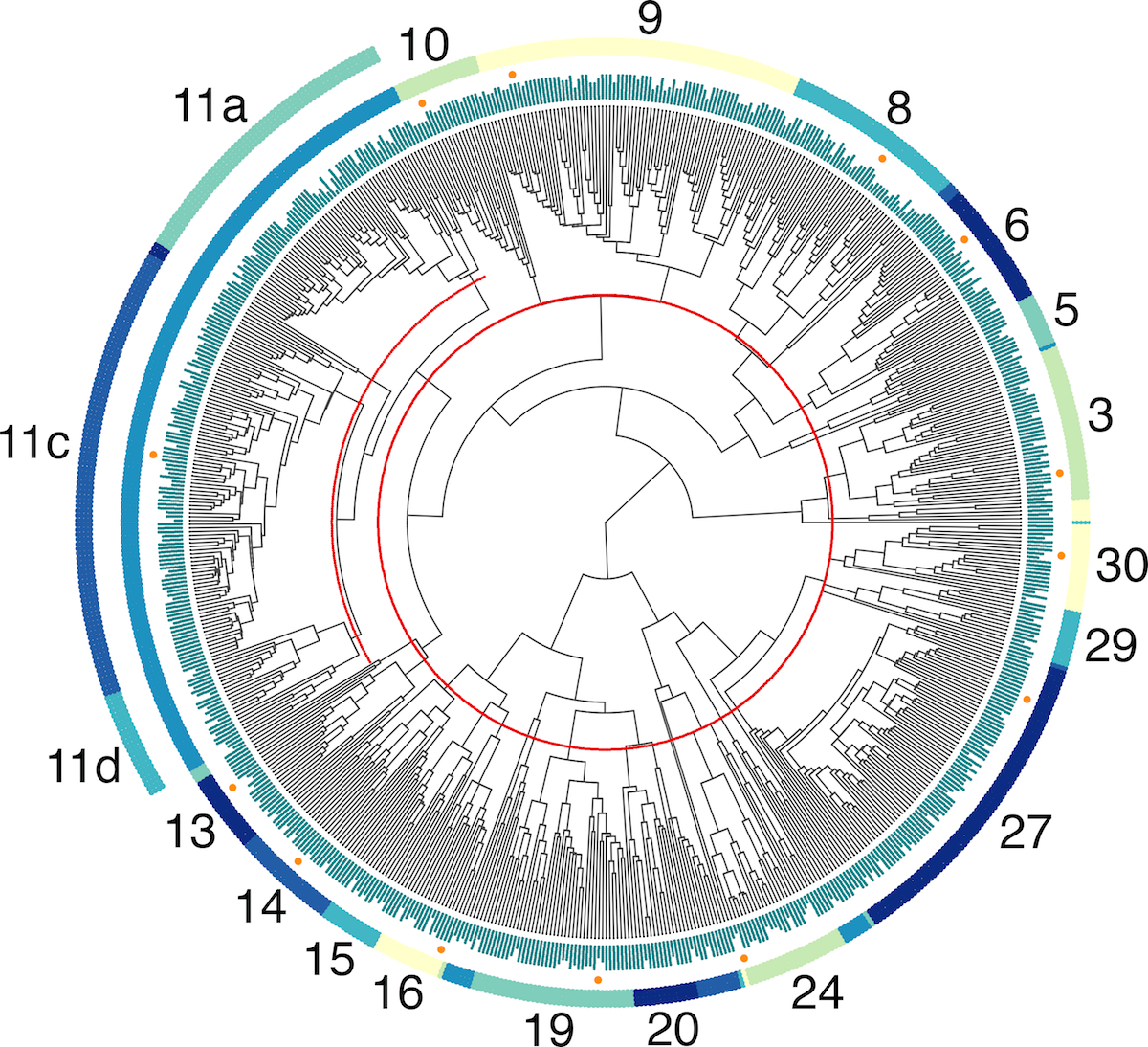
The dependency paths in clusters 14-16 all describe the binding of C to a protein, G, which is usually a receptor for C. In the associated sentences, C is often an endogenous compound, such as an amino acid or hormone.

Clusters 11c and 19-21 contain relationships of reverse directionality from the rest of the dendrogram. The relationships we have described so far relate to situations where the chemical, C, acts on the protein, G, perhaps by inhibiting it, inducing its activity, or raising/lowering its expression/synthesis. Instead, clusters 11c and 19-21 describe situations where the protein acts on the chemical: enzymes that modify chemical structures, transporters that shuttle chemicals across cell membranes, and a variety of other pharmacokinetic (PK) relationships.

Cluster 11c contains most of the PK relationships, including effects of G on C’s metabolism and situations where C is actually a metabolite produced by G after acting on some other chemical. Some transport relationships are also found here, though most of these are in clusters 19 and 21.

Cluster 20 refers to enzymatic modification of C by G. Usually G is an enzyme that specifically targets C and contains C’s name within its own name.

While cluster 11c contained some fine-grained local structure – dependency paths specifically referring to metabolism or secretion tended to cluster close together, for example – it was surprisingly difficult to distinguish different classes of PK relationships within this cluster.

We did not assign themes to the last major group of clusters in the dendrogram (clusters 23-30) because these reflected a major class of errors where part of a protein, such as an amino acid or specific binding domain like a zinc finger, was misidentified as a chemical. While amino acids and elements like zinc are chemicals, the relationships reflected here are whole-part, not interactions between distinct entities.

**­Table A1:** Cluster descriptions for chemical (C) – gene (G) interactions, following the cluster numbers illustrated in Figure 2 in the main text.

| **Cluster Number** | **Cluster Size** | **Theme** | **Selected Descriptive Patterns** | **Entity Pair with Pattern**  **(C / G)** |
| --- | --- | --- | --- | --- |
| 3 | 36 | inhibition | “C, a G inhibitor”  “G specific inhibitor, C”  “C, an inhibitor of G”  “G inhibition by C”  “effects of the G inhibitor, C, on…” | ARRY-614 / p38  naringenin / Smad3  PSC_833 / P-glycoprotein  NVP-AUY922 / Hsp90  SCH_34826 / enkephalinase |
| 5 | 12 | effect on protein activity | “[chemical]-dependent effects of C on G activity”  “effect of C on G activity”  “inhibition of G activity by C”  “study on interaction of C with G”  “G activity in patients on C” | fenfluramine / renin  donepezil / acetylcholinesterase  plumbagin / Nox-4  caffeine / myoglobin  tacrolimus / CYP3A4 |
| 6 | 29 | agonism / antagonism | “effect of C, a selective G antagonist”  “C, a G agonist”  “inactivation of G by C”  “G agonist, C, …”  “study of a G antagonist, C, …” | MTEP / mGluR5  roxindole / 5-HT1A  mitomycin_C / DT-diaphorase  ciglitazone / PPAR-gamma  CI-988 / CCK-B_receptor |
| 8 | 42 | secretion, production, synthesis | “effect of G on C metabolism”  “C inhibits G secretion”  “effects of G on C metabolism”  “G stimulates C production”  “upregulation of C synthesis by G” | dopamine / cholecystokinin  Dasatinib / TNF-alpha  steroid / angiotensin_ii  prostaglandin_E2 / interleukin-1  prostaglandin_E2 / interleukin-1beta |
| 9 | 76 | affects expression | “C inhibits G expression”  “effect of C on G production”  “C induces the expression of G”  “C upregulates G expression”  “effects of C on the expression of G” | AG490 / NFATc1  neopterin / erythropoietin  Nicotine / C-reactive_protein  Dexamethasone / Kv1  letrozole / HOXA10 |
| 10 | 20 | inhibition of activity / expression | “the new G inhibitors, C and…”  “the effect of G inhibition by C”  “inactivation of G by C”  “C effects on G: …”  “the effect of C on G activity in…” | rofecoxib / COX-2  tolcapone / COMT  carbodiimides / thrombin  Naloxone / beta-endorphin  aspartame / acetylcholinesterase |
| 11a | 62 | response to treatment | “G responses to C”  “effect of C on G”  “effect of G with C therapy”  “influence of C on G response”  “effects of C on G release” | cimetidine / Prolactin  gossypol / LDH-X  ribavirin / interferon_alpha_2b  clofibrate / insulin  amines / renin |
| 11c | 96 | metabolism, secretion/uptake | “effect of G on C metabolism”  “effects of G on C formation”  “effect of G on the secretion of C”  “control of G by C”  “G stimulates C uptake”  “[chemical] may reduce G concentration via C”  “G stimulates C transport”  “effect of C on G release” | dopamine / beta-endorphin  cyclic_AMP / adrenomedullin  omeprazole / intrinsic_factor  retinoic_acid / c-jun  phenylalanine / Insulin  catecholamines / leptin  calcium / Prolactin  fenfluramine / growth_hormone |
| 11d | 23 | binding  (uptake/release) | “C release from G”  “binding of C to G”b  “C uptake by G”  “enhancement of action of G by C”  “controlled release of G by C” | iron / transferrin  calcium / troponin_C  potassium / HKT1  glucose / insulin-like_growth_factor_I  polyurethane / IGF-1 |
| 13 | 18 | modulation of expression, substrates | “C modulates [event] through G”  “C binding to G”  “C induced by G”  “C is a G substrate”  “C mediates [event] by G” | Metformin / SIRT1  cyanide / myeloperoxidase  nitric_oxide / iNOS  caffeine / cytochrome_p450_1a2  superoxide / c-Src |
| 14 | 24 | receptor binding | “antagonist of the G C receptor”  “effect of C receptors, G and…”  “[chemical] antagonism of a G C agonist”  “interaction of G with C receptors…”  “a new selective G agonist, C…” | tachykinin / NK1  steroid / pS2  dopamine / D-2  estrogen / DYX1C1  procaterol / beta_2-adrenoceptor |
| 15 | 14 | receptors | “G, a C receptor…”  “deletion of the C G gene…”  “G, a major C receptor…”  “the C domain of G”  “analysis of G C channels…” | free_fatty_acid / GPR40  adenosine / A1_receptor  somatostatin / SSTR4  zinc / SIP1  potassium / KCNQ2 |
| 16 | 16 | receptor [subunit] | “the C carrier subunit (G) of…”  “increased expression of C receptor (G)” “the G subunit of the C receptor”  “human C receptor subunit (G)”  “C receptor G subunits” | acyl / NDUFAB1  benzodiazepine / PBR  NMDA / GluN2B  acetylcholine / CHRNA4  AMPA / GluR1 |
| 19 | 38 | channels / transporters | “regulation of G transporters C and…”  “G is a C channel that modulates…”  “G C transporter expression”  “C transporter, G”  “distribution of G C channel subunits” | sterol / ABCG5  chloride / MOD-1  glucose / GLUT4  glutamate / VGLUT1  potassium / Kv4 |
| 20 | 15 | synthase, dehydrogenase,  reductase | “neuronal C synthase (G)”  “C transporter (G) polymorphism”  “porcine C reductase (G)”  “C dehydrogenase (G)”  “C synthase (G) gene” | nitric_oxide / nNOS  serotonin / 5-HTT  thiol / GILT  Aldehyde / Ald4p  5-aminolevulinate / ALAS1 |
| 21 | 10 | transporters | “G, a C transporter, ...”  “low-affinity C cotransporter (G)”  “a C binding protein, G”  “C transfer protein (G) polymorphism”  “C binding protein (G)” | ribavirin / ENT1  sodium_glucose / SGLT2  methyl_CpG / Mecp2  Cholesteryl_ester / CETP  fatty_acid / hFABP |
| 24 | 24 | sequence, factor, moiety | “complete C sequence of G”  “G is C exchange factor”  “binding of [chemical] to the C moiety of G”  “structural analysis of the C finger of G”  “C binding domains of G” | amino_acid / GSTM4  guanine_nucleotide / Rab3GEP  heme / cytochrome_P-450  zinc / THAP1  nucleotide / CFTR |
| 27 | 74 | phosphorylation / kinases | “expression of receptor C kinase, G”  “G receptor C kinases”  “[gene name] (G) C phosphorylation”  “G C phosphorylation pathway”  “implication of G and C kinase in…” | tyrosine / Trk  tyrosine / ErbB  tyrosine / GIT1  serine / STAT3  creatine / esterase_D |
| 29 | 13 | phosphorylation / phosphatases | “G induces C phosphorylation”  “G C phosphatase”  “C phosphorylation sites on G”  “conserved C residues in G”  “a critical C residue in G” | tyrosine / Oncostatin_M  tyrosine / Shp2  Serine / IRS2  histidine / lipoxygenase  lysine / apolipoprotein_B-100 |
| 30 | 20 | inhibition / activation (via phosphorylation?) | “[other chemical] inhibits C activation of G”  “efficacy of G C kinase inhibitors”  “surface of G C domain”  “discovery of C G inhibitors”  “G induces rapid C phosphorylation” | phenylephrine / phospholipase_A  tyrosine / EGFR  zinc / TFIIB  glycine_hydrazide / CFTR  tyrosine / Prolactin |

*A.2 Chemical-disease relationships*

Here we describe the major clusters in Figure 3 of the main paper (reproduced below). We use "C" to represent a chemical, and "D" to represent a disease.


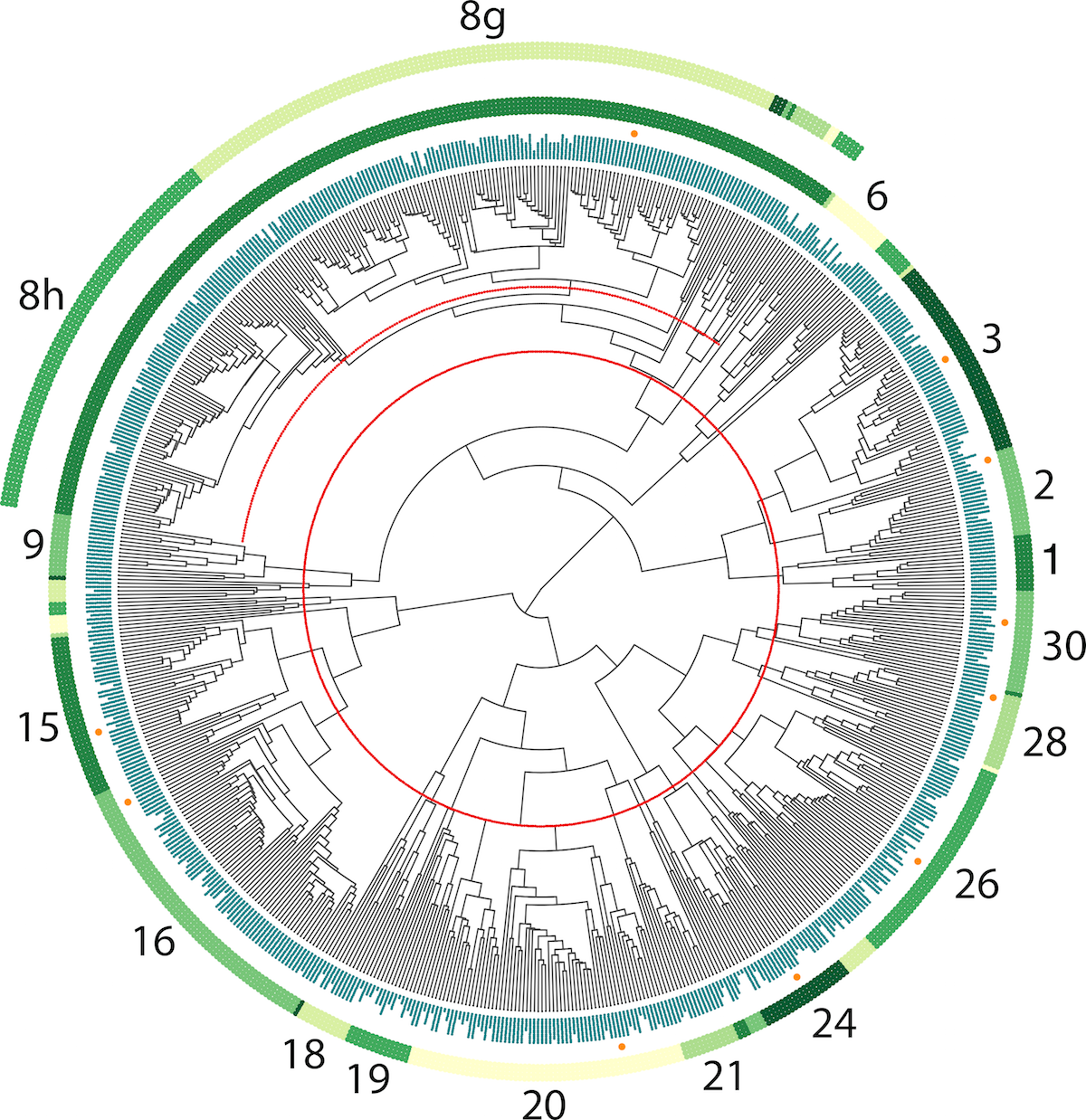
By far the largest set of chemical-disease relationships (from clusters 8g, 8h and 9) are treatment relationships, in which a chemical, C, is described as a treatment or potential treatment for a disease, D. Similar to cluster 3 from Figure 2, these relationships are mostly described in a static context: we don’t know why C is a useful treatment for D, but it is described as such without further elaboration.

While we did not choose to separate clusters 8g, 8h and 9 into different themes, there are subtle differences among these three clusters. Cluster 8g mostly describes evaluation of efficacy; C is investigated as an experimental treatment for D, or patients are described as receiving C for D without indication of whether C is useful. Dependency paths in cluster 8h tend to go further, indicating that the treatment was efficacious for D. Finally, cluster 9, a small cluster with only 14 dependency paths, includes statements about using C to prevent or reduce D, which is slightly different than treating D. However, due to the substantial similarities among these three clusters (some variant of the phrase “treatment for” appears in all three), we labeled all of them with the same theme.

Cluster 6, which also involves the word “treat”, refers mainly to the evaluation of side effects in C-treated patients. Despite its proximity to clusters 8 and 9 in the dendrogram, it is semantically more related to clusters 15 and 16, which describe side effects. In these clusters, D is not a disease that C is used to treat, but a side effect or adverse event resulting from treatment with C.

Cluster 20, which is close in meaning to clusters 15 and 16, includes statements implicating C in the pathogenesis of D. Here C is most often an endogenous compound. Whereas in clusters 15 and 16 we tend to see situations where a drug is intentionally administered to a patient or animal, causing an adverse event, cluster 20 refers to cases where levels of C (most often in serum or tissue) are associated with the risk or progression of D. These levels may result from external supplementation or overproduction of an endogenous compound by the body.

Related to cluster 20 are clusters 18 and 19, which describe biomarkers. In these situations, C is not implicated in the pathogenesis of D, but is instead referred to as an indicator, or marker, of disease progression. There is considerable overlap with the patterns used in cluster 20, but again the shift in meaning is subtle - a substance can be an indicator of D without causing D.

Finally, several clusters relate closely to the concept of disease treatment, but rather than stating “C is a treatment for D”, they instead describe observations about what C is doing. Clusters 1, 9, 21, 24, and 28 all refer to situations where C prevents D, or reduces the risk of D (note that cluster 9 appears both in the “prevents” theme, Pr, and in the “treatment/therapy” theme, T, in Table 2 in the main text). In contrast, clusters 26 and 30 refer to cases where C alleviates D, or reduces its effect. The implication here is that C is being used after D has already occurred.

**Table A2:** Cluster descriptions for chemical (C) – disease (D) interactions, following the cluster numbers illustrated in Figure 3 in the main text.

| **Cluster Number** | **Cluster Size** | **Theme** | **Selected Descriptive Patterns** | **Entity Pair with Pattern**  **(C / D)** |
| --- | --- | --- | --- | --- |
| 1 | 13 | prevents, reduces incidence | “C and [other drug] reduce [adverse event] after D”  “C decreased levels of [substance] after D”  “D of patients treated with C”  “[women, men] receiving C to prevent D”  “intravenous C reduces the incidence of D” | Isoflurane / cerebral_ischemia  estrone / brain_injury  triptans / coronary_spasm  nevirapine / HIV-1_vertical_transmission  magnesium / arrhythmias |
| 2 | 20 | inhibits growth / proliferation | “C significantly inhibited the growth of D”  “C inhibits proliferation of D cells”  “C inhibited [event(s)] in D cells”  “C inhibited D growth”  “C inhibits D growth in vitro” | celastrol / osteosarcoma  Darbepoetin / hepatic_cancer  NVP / RCC  sorafenib / tumor  Zebularine / acute_myeloid_leukemia |
| 3 | 46 | induction of effects in cells, esp. resistance; chemotherapy | “[event] induced by C in D cells”  “C therapy for D”  “C resistance in D”  “D resistant to both C and [other drug]”  “chemotherapy agents like C in D treatment” | fenretinide / neuroblastoma  cisplatin / thoracic_malignancies  Tamoxifen / breast_cancer  imatinib / GIST  doxorubicin / hepatocellular_carcinoma |
| 6 | 15 | treatment evaluations (esp. safety) | “C was measured in patients with D”  “we evaluated the effects of C on D”  “C is indicated for D”  “C administered before/after D reduced [event]”  “treatment of D with C” | Glutamic_acid / ischemic_stroke  diphenidol / chronic_constriction_injury  Bicillin_C-R / streptococcal_infections  nicardipine / coronary_artery_occlusion  sulfasalazine / juvenile_spondyloarthropathies |
| 8g | 125 | treatment of disease (esp. evaluation of efficacy) | “C therapy for the treatment of D”  “patients who received C for treatment of D”  “D patients were treated with C”  “effectiveness of C in D”  “comparison of C and [other drug] in D” | indomethacin / PDA  tigecycline / Acinetobacter_infections  DMSO / amyloid_A_amyloidosis  warfarin / atrial_fibrillation  timolol / angle-closure_glaucoma |
| 8h | 80 | treatment of disease (indication of efficacy) | “C may be useful for the treatment of D”  “evaluate the protective efficacy of C in D”  “C is a promising treatment option for patients with D”  “C is approved for the treatment of D”  “C is commonly prescribed for D” | OPC-18790 / congestive_heart_failure  FTY720 / cerebral_ischemia  bosutinib / CML  anidulafungin / intra-abdominal_abscesses  Colchicine / gout |
| 9 | 14 | treatment of disease (prophylactic) | “C may be used for the prevention of D”  “in [children, patients] with D following C treatment”  “C reduces [event] [during, before] D”  “C prevents [event] [during, in] D”  “C reduces the risk of D by X%” | melatonin / premature_aging  MPH / ADHD  thiazolidinedione_ciglitazone / pneumonia  Mibefradil / atrial_tachycardia  raloxifene / vertebral_fractures |
| 15 | 37 | side effects (association) | “D associated with C therapy”  “the use of C has been associated with D”  “C intake was associated with D”  “incidence of D in patients receiving C”  “D occurred after C” | clozapine / tachycardia  moxalactam / thrombocytopenia  caffeine / shorter_nocturnal_sleep_duration  oxaliplatin / hypersensitivity_reaction  alfentanil / hypotension |
| 16 | 67 | side effects (causal implications) / studies inducing effect | “administration of C resulted in D”  “C induces D”  “D was induced by administration of C”  “D was/were induced by infusion of C”  “patient developed D after receiving C” | vincristine / thrombocytopenia  Taxol / myalgias  lidocaine / Hypotension  ouabain / Cardiac_arrhythmias  ceftaroline / eosinophilic_pneumonia |
| 18 | 12 | potential biomarkers | “C levels of D patients were significantly [lower/higher]…”  “monitoring of C in D rats”  “reduced C in D subjects”  “significant elevations of C in D subjects”  “effect of C on [biomarker level / event] in D patients” | homocycteine / hyperthyroid  homocysteine / hypertensive  selenium / asthmatic  leucine / MSUD  clozapine / schizophrenic |
| 19 | 15 | potential biomarkers | “effect of C supplementation in D”  “we studied the effect of C on D”  “C was well tolerated in [patient group] with D”  “blood C concentrations in patients with D”  “examine the C status of our D patients” | vitamin_D3 / Autism_Spectrum_Disorder  rosiglitazone / angiogenesis  tolterodine / incontinence  vitamin_C / diabetes_mellitus  magnesium / chronic_ambulatory_peritoneal_dialysis |
| 20 | 63 | levels associated with disease risk / progression | “high C levels are associated with increased risk of D”  “C implicated in D”  “effect of D on serum C levels”  “patients with D and increased C concentrations”  “C has been implicated in the pathogenesis of D”  “C intake may be associated with [lower/higher] risk of D”  “C supplementation and incidence of D: …” | cholesterol / coronary_heart_disease  bisphosphonates / osteonecrosis  testosterone / prostate_cancer  triglyceride / unstable_angina  Serotonin / migraine  PUFA / colorectal_neoplasia  beta-carotene / cancer |
| 21 | 13 | changed incidence / risk | “C use was associated with [increased/decreased] risk of D”  “C reduce(s) the risk of D”  “C may reduce the incidence of D in…”  “C was associated with a [lower/higher] risk of D”  “relation of C to risk of D” | Warfarin / ICH  Bisphosphonates / osteoporotic_fractures  Eicosapentaenoic_acid / cardiovascular_disease  Preconception_O3 / GDM  cholesterol / coronary_heart_disease |
| 24 | 22 | inhibits, suppresses | “C inhibited [other event] in D”  “the D action of C”  “C suppresses D through [mechanism]”  “influence of C on D development”  “C significantly suppressed D” | Ki23057 / gastric_tumours  diltiazem / hypotensive  Evodiamine / hyperalgesia  histamine / seizure  AS1069562 / allodynia |
| 26 | 48 | inhibited / blocked disease progression | “the effects of C on the progression of D”  “C may protect against D”  “C blocked D in organ culture”  “C antagonized [other drug-induced] D”  “C attenuates D in mice”  “C ameliorated D by [mechanism]”  “C alleviates D in [disease model]” | minocycline / encephalopathy  Eicosapentaenoic_acid / atherosclerotic_disease  phenethyl_caffeiate / hyperplasia  procyclidine / seizures  Simvastatin / pulmonary_fibrosis  EGB / endothelial_dysfunction  Propentofylline / hypersensitivity |
| 28 | 17 | preventive effects evaluated | “examine the effects of C on D”  “study was carried out to evaluate the effect of C on D”  “investigated possible beneficial effects of C on D”  “to assess the effect of C on D”  “C effective for the prevention of D” | metformin / cytotoxicity  atorvastatin / inflammation  AdCbl / atopic_dermatitis  nebivolol / endothelial_dysfunction  dronedarone / atrial_fibrillation |
| 30 | 23 | reduced, abolished, prevented | “C prevents D”  “C, a [description], prevented D”  “C is beneficial in D”  “D was reduced by C”  “C was effective in reducing D” | Itraconazole / fungal_infections  AMD3100 / anxiety_behaviors  lithium / tauopathies  gabapentin / Pain  buspirone / overall_anxiety_symptoms |

*A.3 Gene-disease relationships*

Here we describe the major clusters in Figure 4 of the main paper (reproduced below). We use "G" to represent a gene, and "D" to represent a disease.

Clusters 2h, 4, 6, 8, and 9 contain relationships that are quite similar to cluster 20 in Figure 3. All of these clusters describe situations where a protein (or chemical, in Figure 2 cluster 20) is implicated in the pathogenesis of a disease. Clusters 4 and 6 refer simply to increased levels of G in D, whereas clusters 8 and 9 more directly implicate the protein in D pathogenesis. Cluster 29 reflects a slightly different theme in which the protein promotes disease progression, rather than disease onset. The two themes share some overlap but are subtly different; cluster 29 focuses on cancers, discussing proteins promoting cell invasion, proliferation, and progression. **
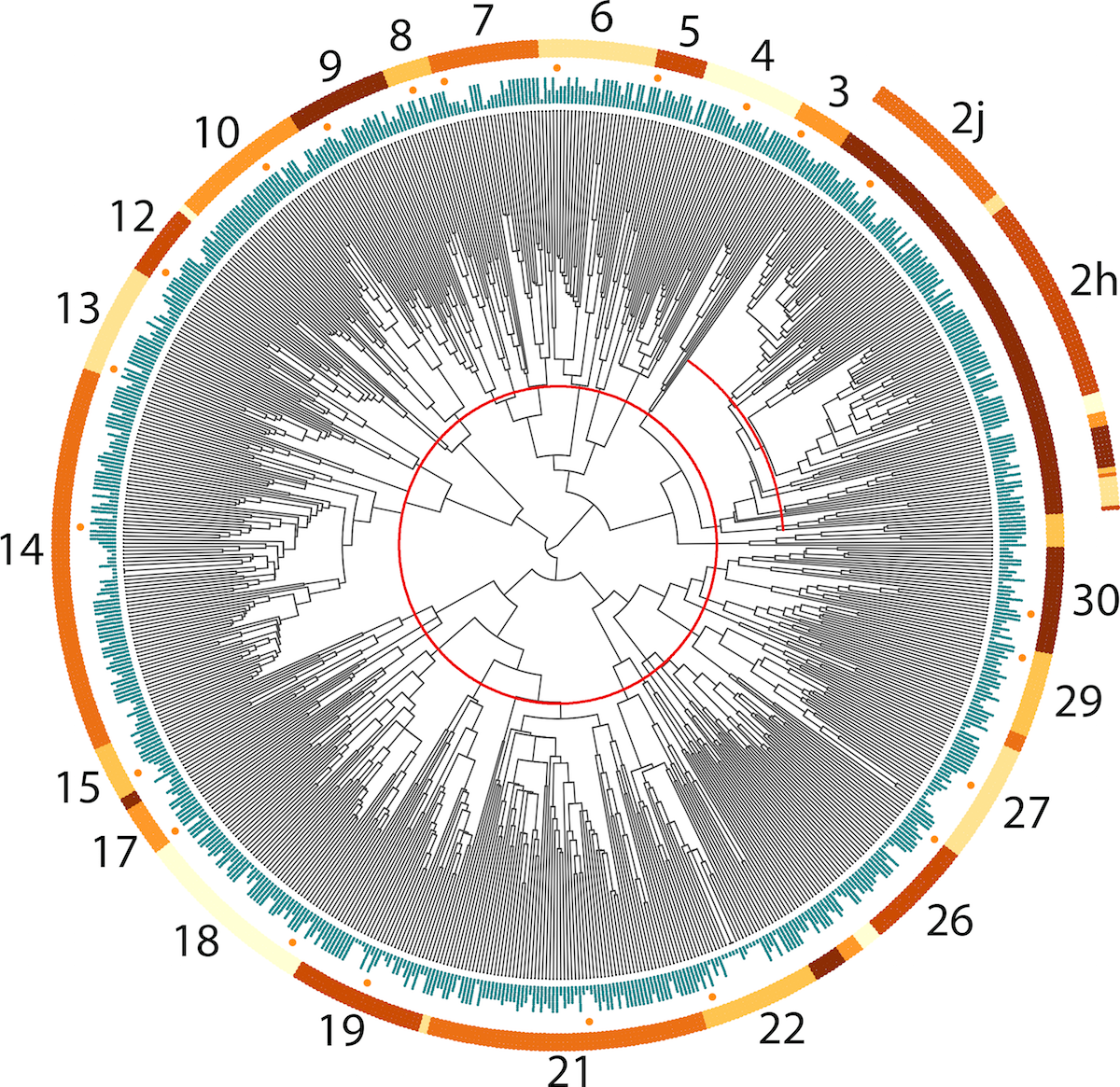
**

Clusters 2j and 3 include therapeutic relationships, where G is described as a treatment or potential treatment of D. Cluster 3 mostly describes trials of G in the treatment of D. While there are a few statements that could perhaps imply efficacy, such as “G therapy for patients with D”, the treatment relationships here are not described with anywhere near the definiteness of clusters 8 and 9 in Figure 3.

Clusters 5 and 7 are similar to clusters 18 and 19 in Figure 3 in that they do not ascribe a pathogenic role to the protein (or chemical) but instead refer to it as a biomarker. Cluster 7 contains statements where a protein, G, is described as “a robust diagnostic biomarker for D”, or “an indicator of D”, without insinuating that it causes D. Cluster 5 is very closely tied to cluster 6, but cluster 6 contains a few statements with causal implications, such as “G is a mediator of D”.

In clusters 10 and 12, the protein, G, is described as a drug target or potential target for the treatment of the disease, D. Often this description does not include the word “target”, but it is implied - the statement refers to the utility of G inhibitors in treating D, for example.

Some statements in clusters 10 and 12 refer to mutations in G that have an effect on D. It’s implied that disruptions in the activity of G can impact the course of D. Clusters 13 and 14 address the issue of mutations more directly, either by describing studies that investigate the role of G mutations in the progression of D (cluster 13) or by directly implicating mutations in G as causal risk factors in D (cluster 14).

While clusters 5 and 7 refer specifically to biomarkers, clusters 15, 17 and 30 refer to overexpression of proteins in disease, usually in patient serum. These proteins could represent potential biomarkers as well, although they are not described in that way.

Clusters 18, 19 and 21 focus on regulation, specifically cases where improper regulation of a gene is linked to disease. There is substantial overlap between these ideas and those of overexpression, biomarkers, etc. but again the focus is subtly different.

The last set of clusters, 22, 26 and 27, focus explicitly on polymorphisms that increase disease risk. The terms “polymorphism”, “mutation”, and “variant” are all present. Cluster 22 focuses almost exclusively on tumor suppressor genes, which, when mutated, can cause cancers. Note that in this case it is mutations in the gene (the DNA) that are increasing risk, rather than the level or activity of a protein. There is some semantic overlap with clusters 13 and 14.

**Table A3:** Cluster descriptions for gene (G) – disease (D) interactions, following the cluster numbers illustrated in Figure 4 in the main text.

| **Cluster Number** | **Cluster Size** | **Theme** | **Selected Descriptive Patterns** | **Entity Pair with Pattern**  **(G / D)** |
| --- | --- | --- | --- | --- |
| 2h | 44 | therapeutic effects, esp. drug sensitivity, resistance | “G and response to [drug] in patients with D”  “G resistance in patients with D”  “serum G levels are associated with D”  “G sensitivity in D”  “comparison of G and [other drug] for detection of D” | TCF7L2 / type_2_diabetes  insulin / systemic_lupus_erythematosus  leptin / hepatic_steatosis  insulin / hypertension  cardiac_troponin_I / ischemic_myocardial_injury |
| 2j | 33 | influences disease treatment (some adjuvant therapies) | “the use of G in the treatment of D”  “D in patients treated with G”  “effect of G on [event] in D patients”  “G therapy in patients with D”  “efficacy of G in D” | parathyroid_hormone / osteoporosis  interferon_alpha_2b / Acute_renal_failure  prolactin / systemic_lupus_erythematosus  Erythropoietin / chronic_renal_failure  S-1 / colorectal_cancer |
| 3 | 13 | therapy, trial, treatment | “G gene therapy of D”  “study of G in D”  “trial of G in the treatment of D”  “relationship between G and [substance] in D patients”  “G treatment for D” | Connexin_26 / bladder_cancer  epidermal_growth_factor / gastric_carcinoma  VP-16 / chronic_granulocytic_leukemia  apolipoprotein_H / stroke  Epoetin / anaemia |
| 4 | 24 | protein causes change in disease status | “injected G induces D”  “G promotes D”  “regulation of [event] by G in D”  “G inhibits D”  “G exacerbates D” | IL-1 / anorexia  VEGF-D / metastasis  TDP-43 / frontotemporal_lobar_degeneration  High-mobility_group_box_1 / ulcer_healing  VDUP1 / bacteremic_shock |
| 5 | 12 | levels / expression in disease | “G levels in D patients”  “expression of G in D”  “increased G levels in patients with D”  “[regulation/function] of G system in D”  “G level in D” | Interleukin-6 / headache  SFRP4 / primary_serous_ovarian_tumours  thyroglobulin / nontoxic_goiter  interleukin-6 / stroke  C-reactive_protein / atopic_dermatitis |
| 6 | 28 | levels / expression in disease | “G levels in patients with D”  “G levels in D patients”  “effects of [drug] on G in D patients”  “serum G levels in D”  “expression of G in D” | interleukin-6 / glomerulonephritis  Interleukin-2 / multiple_sclerosis  insulin / hypertensive  E-selectin / Kawasaki_disease  E-cadherin / carcinomas |
| 7 | 26 | biomarkers, diagnostic | “G is a robust diagnostic biomarker for D”  “G is an independent predictor of D”  “G as an indicator of D in patients with…”  “prognostic significance of G in D patients”  “effects of [situation/event] on G levels in D”  “G is a potential marker of D” | TLE1 / synovial_sarcomas  Proinsulin / coronary_heart_disease  Plasma_hyaluronidase / atherosclerosis  TGFbeta-1 / breast_cancer  chromogranin-A / neuroendocrine_tumors  SERPINA3 / preeclampsia |
| 8 | 11 | role in pathogenesis | “association of G with [event] in patients with D”  “effects of G on D”  “role of G in the development of D”  “role of G in the pathogenesis of D”  “a novel gene, G, is associated with D” | FCGR2A / rheumatoid_arthritis  interleukin-5 / acute_myeloid_leukemias  IL-4 / transplant_arteriosclerosis  leptin / thyroid_cancer  THSD7A / obesity |
| 9 | 24 | role in disease course / pathogenesis | “clinical impact of circulating G in D”  “G attenuates D”  “G predicts [event] in patients with D”  “evidence for role of G in D”  “G: the link between D and [other disease]” | miR-18a / oesophageal_squamous_cell_carcinoma  Wnt5a / pulmonary_arteriolar_remodeling  LTBP2 / acute_dyspnoea  BRCA1 / gastric_cancer  HMGB1 / diabetes_mellitus |
| 10 | 32 | inhibitors used as therapies | “G inhibitors in D: …”  “D with G mutation(s)”  “response to G inhibitors in patients with D”  “G testing and management of D”  “G gene amplification in D” | ACE / aortic_stenosis  TARDBP / amyotrophic_lateral_sclerosis  EGFR / squamous_cell_carcinoma  EGFR / NSCLC  c-erbB-2 / nasopharyngeal_carcinoma |
| 12 | 17 | drug targets (esp. cancer) | “G signaling in D cells”  “G inhibitors in the treatment of D”  “G as a strategic target in D therapy”  “G: an attractive target for D therapy”  “[drug]: a C inhibitor for the treatment of D” | Akt / colon_cancer  MEK1/2 / malignancies  ErbB1 / breast_cancer  Angiopoietin-2 / tumor  tumor_necrosis_factor_alpha / rheumatoid_arthritis |
| 13 | 26 | evaluation of role of mutations in disease | “G mutations in D”  “mutations in G in D”  “characterization of G expression in D”  “G mutations are associated with [event] in D”  “role of G in D development” | KRAS / lung_adenocarcinoma  GUSB / mucopolysaccharidosis_VII  MUC1 / papillary_thyroid_carcinoma  KRAS / colorectal_cancer  RSK2 / osteosarcoma |
| 14 | 91 | causal mutations | “mutation of G in a patient with D”  “G mutation is associated with D”  “novel mutation in G gene associated with D”  “characterization of G mutations causing D”  “mutations of the G gene in patients with D”  “D: a novel G mutation…”  “D: novel G mutations and…”  “the recurrent mutation of G in C patients”  “G mutations can cause D” | STK11 / Peutz-Jeghers_syndrome  MTHFR / arterial_stroke  MYH7 / distal_myopathy  GALC / Krabbe_disease  COL1A2 / osteogenesis_imperfecta  CISD2 / Wolfram_syndrome  NPC1 / Niemann-Pick_type_C_disease  BRCA1 / breast_cancer  HIBCH / Leigh-like_disease |
| 15 | 13 | levels, concentrations, expression | “G levels in patients with D”  “serum G concentrations in D”  “G expression in D cell lines”  “diagnostic value of G in D patients”  “prognostic relevance of G in D” | renin / thoracic_neuroblastoma  leptin / hyperinsulinemia  TIMP-1 / prostate_tumor  interleukin_17 / lung_cancer  CCN3 / Ewing_sarcoma |
| 17 | 12 | levels, overexpression | “serum G concentrations in patients with D”  “serum G level in patients with D”  “G overexpression in D”  “G is overexpressed in D”  “G expression in D patients” | erythropoietin / anemia  thyroglobulin / subacute_thyroiditis  cyclin_D3 / follicular_thyroid_carcinoma  FOXG1 / hepatoblastoma  SPARC / pancreatic_cancer |
| 18 | 43 | expression, mutations correlated with disease | “presence of G gene mutation in D patients”  “frequency of G mutations in D”  “association of D with G mutations”  “association of G expression with D”  “correlation between G expression and [event] in D” | BRAF / melanoma  PTEN / thyroid_cancer  PDH / cerebral_dysgenesis  FcRn / lung_abnormalities  COX-2 / colon_cancer |
| 19 | 32 | gene expression, regulation | “down-regulation of G in D cells”  “expression of G mRNA in D”  “mRNA expression of G in patients with D”  “D cells expressing G”  “regulation of G expression in D cells” | E-cadherin / breast_cancer  CerbB-2 / nasopharyngeal_carcinomas  KCNQ1 / long_QT_syndrome_type_1_and_2  P-gp / acute_myeloid_leukemia  CYP1A1 / medulloblastoma |
| 21 | 66 | gene expression in cell lines | “G expression in D”  “G expression in patients with D”  “analysis of G expression in D”  “effects of G on D cells”  “G expression in D cells” | c-mpl / hematologic_disorders  trypsinogen-1 / ulcerative_colitis  SLC34A2 / ovarian_tumors  p53 / hepatocellular_carcinoma  MMP2 / prostate_cancer |
| 22 | 28 | tumor suppressor genes | “G as a D suppressor”  “G acts as a D suppressor”  “the gene G is a functional D suppressor”  “G, a novel D suppressor”  “G: a mediator of D” | Caspase-2 / tumour  ECRG4 / tumor  GADD45G / tumor  SynCAM / tumor  P-glycoprotein / melanoma_invasion |
| 26 | 26 | polymorphism | “association of variants of G with D”  “association of the G polymorphisms with D”  “genetic polymorphisms at G are associated with D”  “mutations in the G gene in patients with D”  “G polymorphisms are associated with D” | factor_V_Leiden / thrombosis  interleukin-18 / type_1_diabetes  SIRT1 / carotid_atherosclerosis  P-protein / encephalopathy  Chromogranin_A / hypertensive_renal_disease |
| 27 | 28 | polymorphism | “association of G gene polymorphism with D”  “polymorphism of G in D”  “mutation of the G gene in D”  “G polymorphism is associated with D”  “mutation in the G gene in a family with D” | vascular_endothelial_growth_factor / colon_cancer  angiotensin-converting_enzyme / sarcoidosis  endothelin-3 / Waardenburg-Hirschsprung_disease  tumor_necrosis_factor_a / cystic_fibrosis  connexin_32 / Charcot-Marie-Tooth_neuropathy |
| 29 | 20 | promotes progression (cancers) | “G promotes D cell invasion”  “G promotes D cell proliferation”  “expression of G in [disease] correlates with D”  “G promotes D progression by…”  “G expression is associated with D in [disease]” | DLK1 / lung_cancer  CD97 / gastric_cancer  Apaf-1 / lymph_node_metastasis  HDAC6 / hepatocellular_carcinoma  Gli-1 / lymph_node_metastasis |
| 30 | 25 | overexpression associated with disease (cancers) | “regulation of G gene expression in D”  “prognostic value of G in D”  “secretion of G by D in vitro”  “G overexpression in D”  “correlation between G expression and D” | CD44 / neuroblastoma  Gli-1 / gastric_cancer  cathepsin_B / gliomas  TRIB1 / acute_myeloid_leukemia  p27Kip1 / esophageal_squamous_cell_carcinoma |

*A.4 Gene-gene relationships*

Here we describe the major clusters in Figure 5 of the main paper (reproduced below). We use "G1" to represent the first gene, and "G2" to represent the second gene.

The cluster themes in Figure 5 were the most difficult to parse among all the dendrograms. The vast majority of protein-protein relationships reflect some kind of change in activity or expression in the second protein based on the action of the first protein. Many of the relationships are similar to chemical-gene relationships in that a protein binds to another protein (cluster 10), increases its expression (clusters 21 and 22), or affects its expression in some other way that is not stated (clusters 7 and 17). All of these themes also appear in Figure 2.

However, there are a few other themes that are specific to protein-protein interactions. One protein can enhance the response of another to some stimulus (cluster 13), or activate or stimulate another protein by itself (clusters 14 and 16). A protein can be produced by a cell population expressing another protein, as in the case of lymphocytes (i.e. proteins produced by CD4-bearing T-cells), which is reflected in clusters 1, 2 and 6.

Clusters 24, 25, 28 and 30 all reflect similar relationships involving regulation and pathways, but are subtly different. Cluster 24 explicitly refers to signaling, with both protein members forming part of the same signaling pathway. Cluster 25 is a cluster of patterns reflecting abbreviations, where the two proteins involved are literally identical or part of the same protein complex. Clusters 28 and 30 speak more specifically of regulation, but contain several patterns that also refer to co-membership in the same pathway. All of these concepts are related.


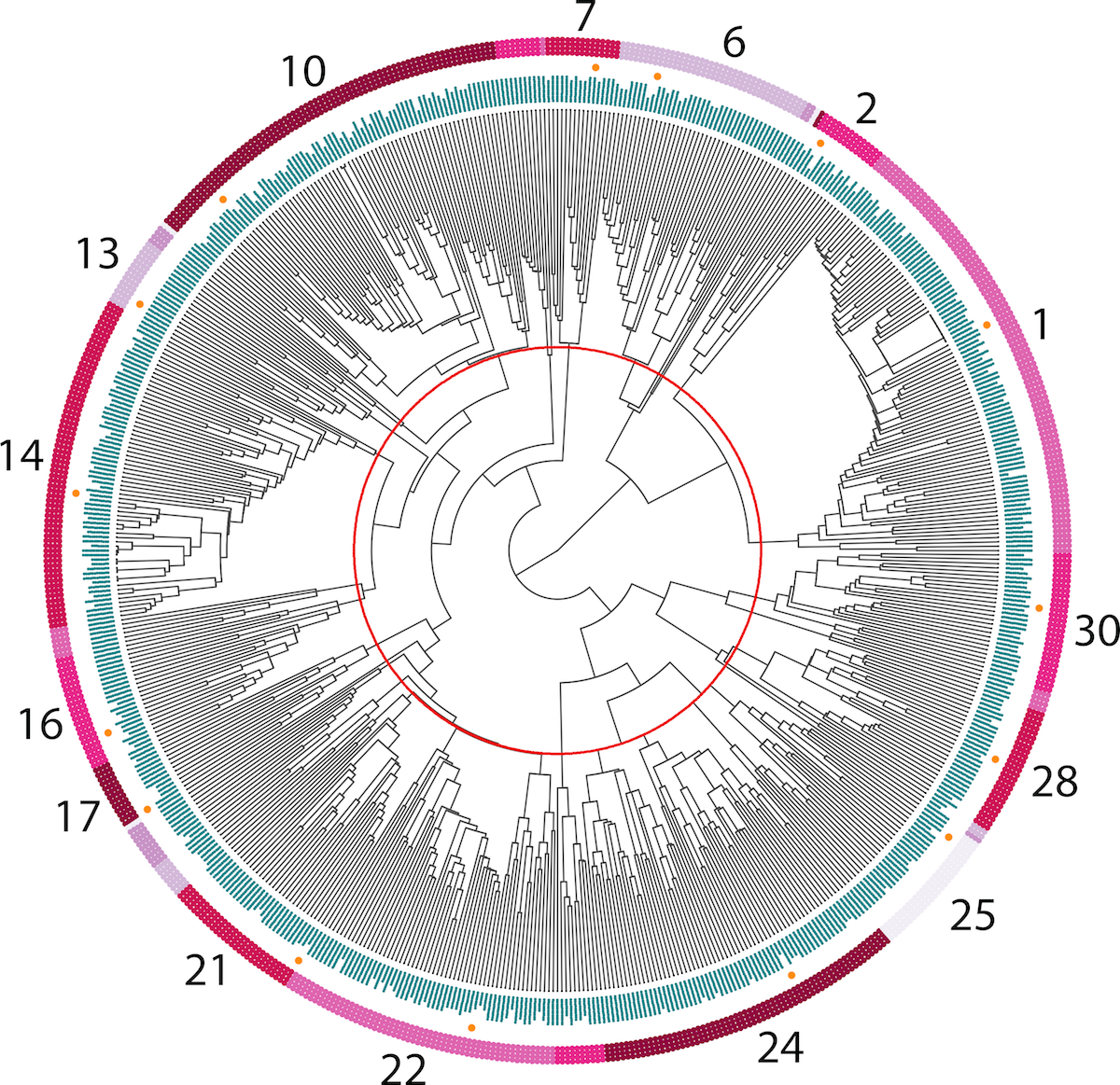


**Table A4:** Cluster descriptions for gene (G1) – gene (G2) (usually protein-protein) interactions, following the cluster numbers illustrated in Figure 5 in the main text.

| **Cluster Number** | **Cluster Size** | **Theme** | **Selected Descriptive Patterns** | **Entity Pair with Pattern**  **(G1 / G2)** |
| --- | --- | --- | --- | --- |
| 1 | 90 | cell populations | “G1 induction of human G2”  “increased induction of G2 in G1 lymphocytes”  “G1 + G2 T-cell population”  “G2 induction of G1”  “G1 expression [on, in] G2 T-cells”  “an enriched G1 + G2 T-cell subset”  “G1-dependent G2 activation” | C5a / interleukin_1  CD8 / interferon-gamma  CD25 / Foxp3  NfkappaB / Interleukin-1beta  CD161 / CD8  CD4 / CD8beta  ERK / CREB |
| 2 | 14 | cell populations, regulation | “regulation of G2 expression by G1”  “G1 induces G2 gene transcription”  “regulation of G2 by G1”  “G2 expression in the G1 + cells”  “G1 / G2 ratio” | SOX10 / MITF  TNF-alpha / MUC1  RECK / matrix_metalloproteinase-9  CD34 / Bcl-2  CD39 / CD8 |
| 6 | 39 | cell populations, protein production / gene expression | “G1 production by G2 + T cells”  “G1 producing G2 + T cells”  “G1 signaling in G2 + T cells”  “G1 expression on G2 + T cells”  “the role of G1 in the function of G2 + T cells” | IL-17A / CD146  IL-10 / CD8  IFN-gamma / CD4  CXCR3 / CD8  CD28 / CD25 |
| 7 | 15 | inhibits / induces expression | “G1 induces G2 expression”  “G1 inhibits G2 expression”  “effect of G2 on G1 production”  “G1 secretion in G2 cells”  “G1 induced G2 production” | Fos / Neurotensin  IL-15 / IL-7Ra  MMP-9 / calcitonin-gene-related_peptide  cholecystokinin / STC-1  TNF-alpha / TARC |
| 10 | 76 | binding, regulation of activity | “G1 binds G2”  “G2 interaction with G1”  “G1 is a receptor for G2”  “G1 binding to G2”  “G1 mediates activation of G2” | HJURP / CENP-A  Bcl-xL / Clusterin  CD96 / CD155  Haptoglobin / apolipoprotein_A-I  Bcl10 / NF-kappaB |
| 13 | 14 | enhances response (esp. hormones) | “G1 enhances [event] via G2”  “changes in the G1 response to G2”  “G1 and G2 responses to [event]”  “G1 in G2 receptor signaling”  “exaggerated G2 response of G1” | Glypican-4 / insulin_receptor  prolactin / thyrotropin-releasing_hormone  Prolactin / TRH  Fc_gamma_RI / p72syk  thyrotropin-releasing_hormone / prolactin |
| 14 | 67 | activation, stimulation, signaling | “G2 activates [protein] via G1”  “G1 stimulates G2”  “G1 modulates G2 signaling”  “G2 stimulates G1 expression”  “G1 induces phosphorylation of G2” | fucosyltransferase_1 / Calreticulin  Akt / SREBP1c  Hsp27 / p53  EGFR / MUC1  Thrombopoietin / STAT5 |
| 16 | 23 | activation, targeting | “function of G2 in G1 receptor activation”  “G2 promotes [event] by targeting G1”  “G1 phosphorylation by G2”  “role of G1 in the activation of G2”  “regulation of G1 expression by G2” | TNFR1 / Ubc13  EPB41L3 / miRNA-223  NuMA / CDK1  PP4 / JNK-1  FGF8 / androgen_receptor |
| 17 | 13 | affects production (mostly induces) | “G2 induces the production of G1”  “[protein] stimulates G2 production via G1”  “regulation of G2 production by G1”  “downregulation of G2 by G1”  “enhancement of G2 by G1” | IgG1 / IL-27  ERK1/2 / granulocyte_colony-stimulating_factor  IFN_gamma / IL-18  miR-25 / mitochondrial_calcium_uniporter  TNF-alpha / IFN-gamma |
| 21 | 28 | induces expression / production | “G2 induces G1 production”  “G1 modulates G2 expression”  “induction of G1 expression by G2”  “G2 upregulates G1 expression”  “G1 stimulates G2 secretion in [cell type] cells” | beta-defensin-2 / Tat  Stat3 / heat_shock_27kDa_protein  iNOS / IL-1beta  p16INK4a / p33ING1b  Angiotensin_II / endothelin-1 |
| 22 | 56 | induces release / production | “G1 induces G2 expression”  “G2 stimulates G1 secretion”  “G1 stimulates G2 release”  “G2 stimulates G1 production”  “effect of G2 on G1 secretion” | CXCL12 / connective_tissue_growth_factor  atrial_natriuretic_peptide / Thrombin  Bradykinin / tissue_plasminogen_activator  MCP-1 / Angiotensin_II  renin / neuropeptide_Y |
| 24 | 62 | signaling, receptor binding | “G2 signaling via G1”  “G1 / G2 costimulatory interactions”  “coactivator G1 in G2 transcriptional activation”  “G2 G1 signaling”  “the G2 G1 receptor”  “binding of G2 to the G1 receptor” | SMOC-1 / TGF-beta  ICAM-1 / LFA-1  CBP / p53  TCF / beta-catenin  TNF / p55  interleukin-1 / interleukin-18 |
| 25 | 26 | same or related protein: abbreviations | “G1 (G2) inhibitor”  “expression of G1 (G2) protein”  “G2 (G1) activity”  “G2 (G1) expression”  “G1 / G2 complexes” | mammalian_target_of_rapamycin / mTOR  pentraxin_3 / PTX3  PON1 / paraoxonase-1  AURKA / Aurora_kinase_A  PAI-1 / vitronectin |
| 28 | 26 | regulation of expression / activity | “the roles of G1 / G2 in [event]”  “G2 (G1) expression”  “binding of G1 / G2 proteins”  “G2 regulates G1 activity”  “synergistic effect of G1 / G2” | MMP-2 / TIMP-2  M-CSF / macrophage_colony-stimulating_factor  NF-kappa_B / Rel  RhoA / Shp-2  IL-6 / BSF-2 |
| 30 | 28 | regulation of expression / activity | “upregulation of G2 activity by G1”  “regulation of G1 expression by G2”  “G1 regulation of G2”  “G2 regulation by the G1 pathway”  “prognostic significance of G1, G2, …” | CD28 / interleukin-4  TNF-alpha / TGF-beta  miR-133b / Connective_Tissue_Growth_Factor  JNK / ATF2  bcl-2 / PCNA |

­
